# Supplementary material for: RNAprecis: Prediction of full-detail RNA conformation from the experimentally best-observed sparse parameters
Source: PLoS Comput Biol. 2026 May 8;22(5):e1014242. doi: 10.1371/journal.pcbi.1014242 (PMC13178996; doi:10.1371/journal.pcbi.1014242)
Supplement: S1 Text — (PDF) [file pcbi.1014242.s001.pdf]

Supplement to RNAprecis: Prediction of full-detail RNA conformation from the experimentally best-observed sparse parameters

Henrik Wiechers[1◑], Christopher J. Williams[2◑], Benjamin Eltzner[1◑], Franziska Hoppe[1], Michael G. Prisant[2], Vincent B. Chen[2], Ezra Miller[3], Kanti V. Mardia[4], Jane S. Richardson[2*], Stephan F. Huckemann[1**]

April 23, 2026

1 Felix-Bernstein-Institute for Mathematical Statistics in the Biosciences, University of Göttingen, Göttingen, Germany
2 Department of Biochemistry, Duke University School of Medicine, Durham, North Carolina, United States of America
3 Mathematics Department, Duke University, Durham, North Carolina, United States of America
4 Department of Statistics, University of Leeds, Leeds, United Kingdom
◑ These authors contributed equally to this work.
* jsr@kinemage.biochem.duke.edu
** Stephan.Huckemann@mathematik.uni-goettingen.de

Contents

Appendices 2

A High detail training data set (HD) 2

B Low detail test data sets 5
- B.1 LDT test data . . . 5
- B.2 8b0x test data . . . 7

C Clustering results of HD and comparison with suitename 9

D Low detail (LD) parameter space 18

E Results RNAprecis 22
- E.1 Results for the test data set LDTP33 . . . 22
- E.2 Results for the test data set LDTP32 . . . 25
- E.3 Results for the test data set LDTP23 . . . 28
- E.4 Results for the test data set LDTP22 . . . 31
- E.5 Results for the test data set 8b0xP33 . . . 33
- E.6 Results for the test data set 8b0xP32 . . . 37
- E.7 Results for the test data set 8b0xP23 . . . 40
- E.8 Results for the test data set 8b0xP22 . . . 42

Appendices

A High detail training data set (HD)

This section introduces in Table A the PDB files with the corresponding resolution of the high resolution training data set (introduced in Section 2.1), from which the HD and LD data sets (and the corresponding four pucker-pair sets) are composed. In Fig A the four pucker-pair sets of HD are plotted and in Fig B the four sub-data sets of LD are plotted.

PDB	Res	PDB	Res	PDB	Res	PDB	Res
2jLv	1.90	3t5n	1.79	4z4d	1.60	6cmn	1.80
2v7r	1.20	3td0	1.60	4zLd	1.60	6d92	1.81
2xLk	1.80	3zp8	1.55	5L00	1.25	6db8	1.87
2xs7	1.45	4Lgt	1.30	5L2L	1.55	6dtd	1.65
2y8w	1.80	4Lvw	1.77	5Lr3	1.65	6du4	1.70
2y8y	1.44	4aLp	1.48	5Lr4	1.80	6e1w	1.69
2zko	1.70	4d25	1.90	5ay3	1.20	6e8u	1.55
2zy6	1.75	4fen	1.35	5ay4	1.70	6f4g	1.90
3La5	1.70	4gv6	1.98	5b2q	1.70	6gc5	1.90
3bso	1.74	4j39	1.70	5c5w	1.25	6gd2	1.90
3c3z	1.50	4j50	1.65	5da6	1.05	6h0r	1.73
3cgp	1.57	4jah	1.50	5dhb	1.80	6h9h	1.75
3cgs	1.65	4jrd	1.00	5eLh	1.80	6hau	1.86
3d2s	1.70	4k31	1.42	5eim	1.54	6hc5	1.41
3dd2	1.90	4mcf	1.90	5ev4	1.57	6i0v	1.85
3diL	1.90	4mdx	1.50	5ew4	1.47	6iv9	1.86
3gLp	1.23	4ms9	1.32	5fjc	1.71	6jjh	1.74
3gvn	1.20	4n0t	1.70	5gjb	1.70	6kwq	1.76
3h5y	1.77	4nLf	1.00	5h1k	1.90	6oon	1.90
3i62	1.95	4p3t	1.60	5hby	1.18	6ozn	1.90
3iev	1.90	4p97	1.86	5jaj	1.50	6ppq	1.81
3jxq	1.45	4pcj	1.90	5jrc	1.90	6q1h	1.45
3nd3	1.37	4pco	1.32	5kLa	1.14	6qit	1.50
3nd4	1.52	4qpx	1.86	5mwi	1.51	6sce	1.83
3nmr	1.85	4qvi	1.90	5nxt	1.38	6tqb	1.60
3oin	1.90	4rbq	1.05	5udi	1.58	6u8d	1.81
3pdr	1.85	4rby	1.19	5v3f	1.70	6ugg	1.95
3pey	1.40	4rc0	1.13	5vj9	1.57	6ugi	1.75
3qgc	1.90	4rne	1.01	5wLh	1.80	6uv4	1.70
3r1d	1.45	4u3L	1.48	5www	1.80	8a3d	1.67
3rer	1.70	4wan	1.80	6b14	1.64	8b0x	1.55
3sj2	1.36	4xw0	1.81	6cb3	1.89		
3syw	1.57	4xwf	1.80				

Table A: PDB IDs (sorted in alphabetical order) and resolutions of the different measurements of the HD data set (introduced in Section 2.1 in the main text)

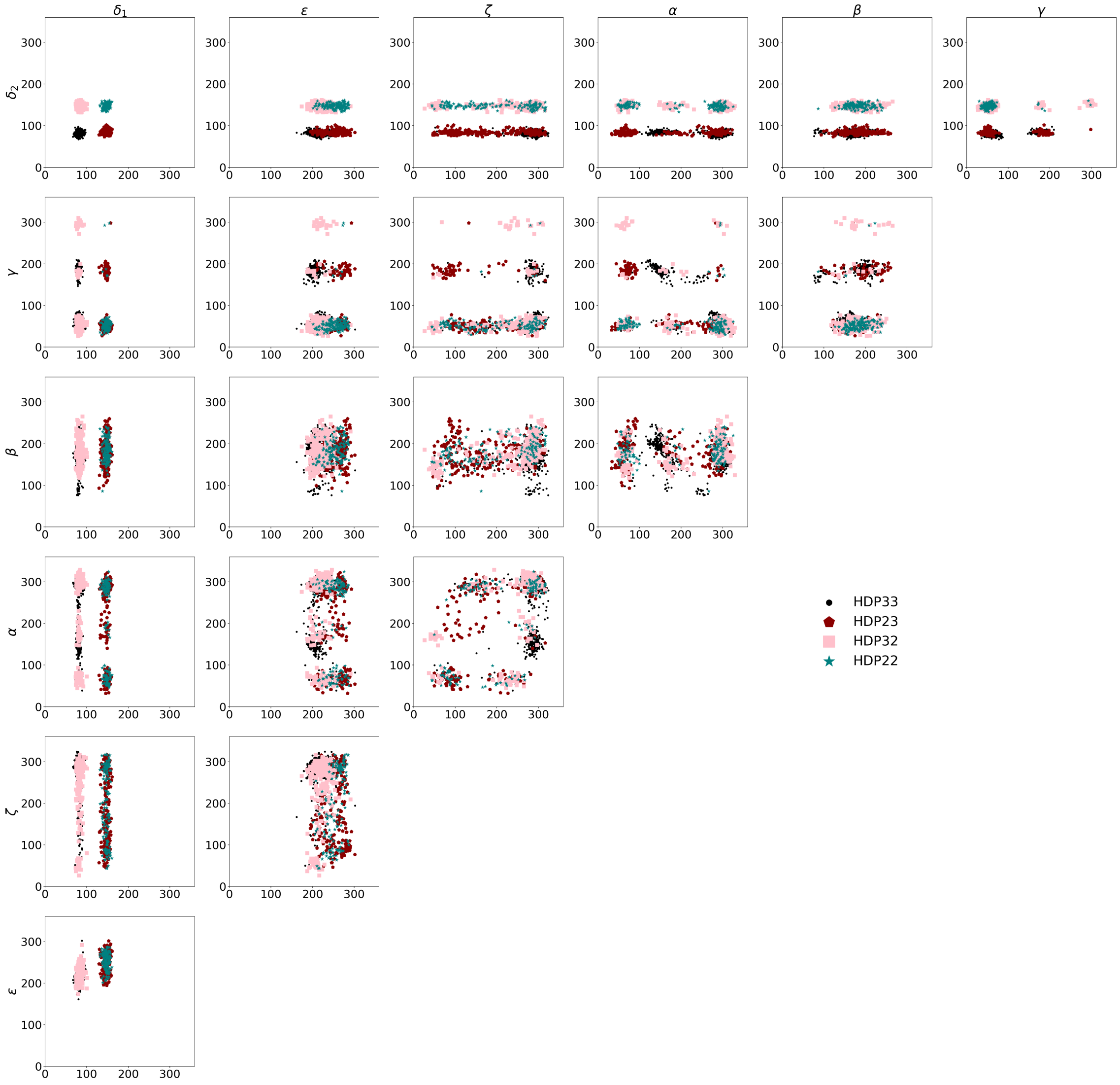

Fig A: The HD representation of the high resolution training data set, resolved in pucker-pair (introduced in Section 2.1 in the main text), represented by scatter plots of all two-dimensional dihedral angle pairs.

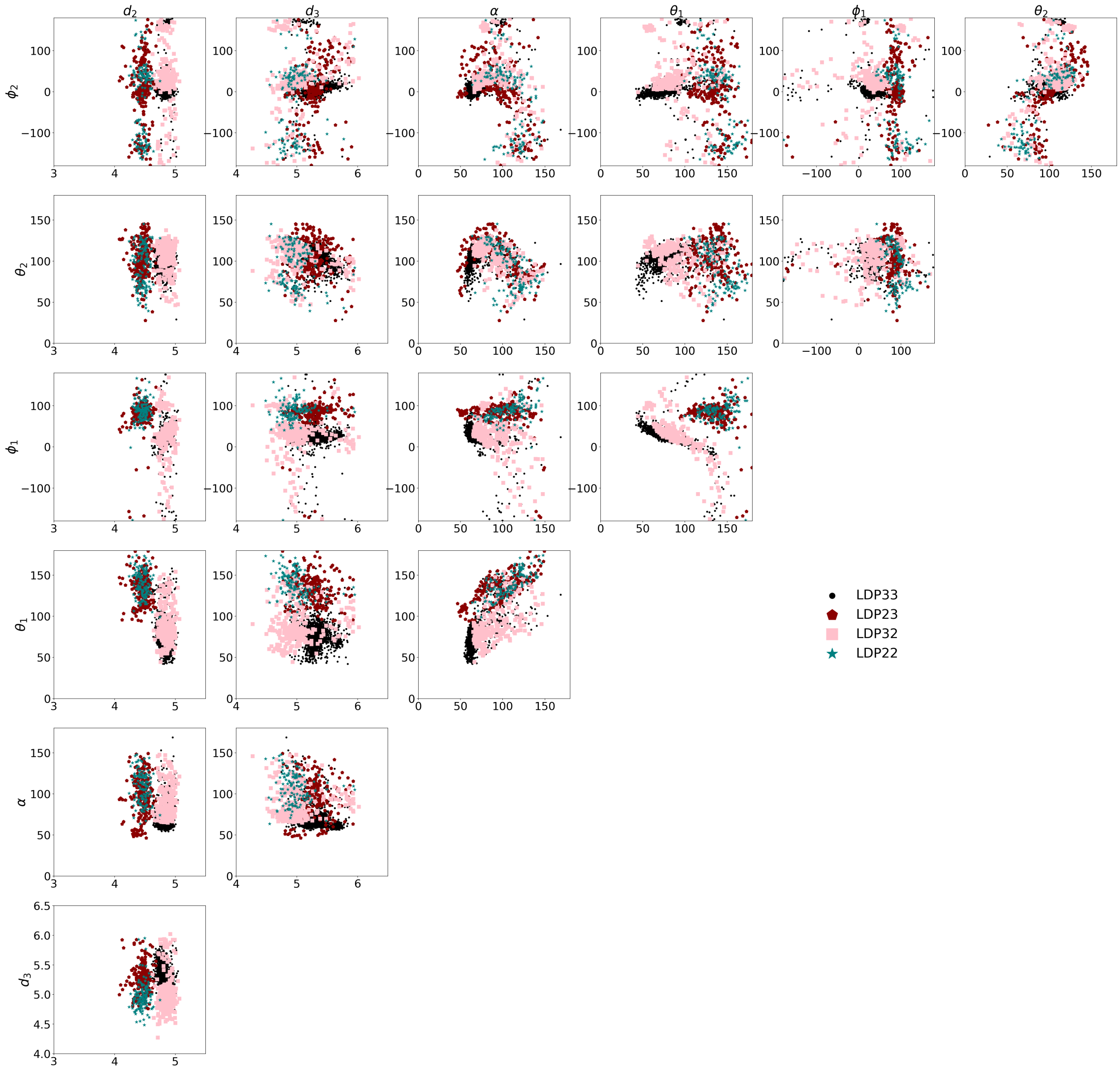

Fig B: The LD representation of the high resolution training data set, resolved in pucker-pair (introduced in Section 2.1 in the main text), represented by scatter plots of all combinations of parameters (described in Section 2.3.1 in the main text).

B Low detail test data sets

B.1 LDT test data

Table B lists the suites contained in the LDT test data set (introduced in Section 2.1). In Fig C the four pucker-pair sets of LDT are plotted.

PDB	suite name (chain-residues)	res
1c0a	B-634_635, B-636_637	2.4
2eet	A-17_18, A-19_20, A-20_21	1.95
”	A-44_45	”
2eev	A-26_27, A-61_62, A-79_80	1.95
2qbz	X-29_30, X-34_35, X-35_36	2.6
”	X-45_46, X-47_48, X-85_86	”
”	X-86_87, X-87_88, X-100_101	”
”	X-101_102, X-113_114, X-125_126	”
”	X-138_139, X-144_145, X-154_155	”
”	X-170_171, X-171_172	”
2xli	B-16_17	2.33
3bsn	T-5_6, T-6_7	1.8
3ger	A-15_16, A-48_49, A-53_54	1.7
”	A-62_63, A-63_64	”
3gx5	A-9_10, A-15_16, A-18_19	2.4
”	A-19_20, A-23_24, A-29_30	”
”	A-35_36, A-52_53, A-63_64	”
”	A-68_69	”
3i5x	B-3_4	1.9
3oij	C-2_3	3.0
3q51	A-30_31	2.85
3t5q	L-4_5	3.0
3u4m	B-2115_2116	2.0
3u56	B-2182_2183	2.1
3umy	B-2144_2145, B-2181_2182	1.9
4fnj	A-3_4, A-4_5, A-30_31	1.95
4kze	R-61_62	2.4
4lvv	A-81_82	2.1
4lvy	A-54_55	2.0
4qg3	B-2125_2126, B-2172_2173	2.0
4u3p	B-6_7	1.87
4w5t	B-8_9, B-12_13	2.5
4xw7	A-24_25, A-27_28	2.5
4yn6	A-2_3, A-9_10, B-1_2	2.3
4z4g	B-15_16, B-16_17, D-7_8	2.7
5b2o	B-17_18	1.7
5dhb	C-1_2, C-2_3	1.8
5fk1	A-16_17, A-20_21, A-46_47	2.5
”	A-50_51, A-56_57, A-67_68	”
”	A-90_91	”
5neo	A-7_8	1.69
5npm	B-3_4, B-7_8, B-14_15	2.7
6b3k	R-28_29, R-57_58	2.09
6d8f	H-10_11, H-16_17	2.15
6h9i	E-8_9, E-12_13	2.29
6q57	A-34_35, A-53_54, A-82_83	2.72
”	A-87_88	”
6qis	G-5_6	1.99
6u8k	C-157_158, C-160_161, C-201_219	2.75
6xh0	D-35_36	3.1
6xh1	D-33_34	2.6

PDB	suite name (chain-residues)	res
6y0t	A-2647_2648, A-2659_2660	1.39
7e9i	A-25_26, A-31_32	2.8
7eem	A-2652_2653, A-2660_2661	2.59
7f5s	C-16_17, C-55_56, C-151_152	2.72
”	C-349_350, C-940_941, C-976_977	”
”	C-1393_1394, C-1524_1525, C-1585_1586	”
”	C-1620_1621, C-1639_1640, C-1640_1641	”
”	C-1641_1642, C-1881_1882, C-1888_1889	”
”	C-2401_2402, C-2411_2412, C-2464_2465	”
”	C-2510_2511, C-2737_2738, C-2738_2739	”
”	C-2741_2742, C-2815_2816, C-2834_2835	”
”	C-3684_3685, C-3747_3748, C-3869_3870	”
”	C-3884_3885, C-3925_3926, C-4221_4222	”
”	C-4224_4225, C-4331_4332, C-4337_4338	”
”	C-4354_4355, C-4379_4380, C-4390_4391	”
”	C-4465_4466, C-4487_4488, C-4498_4499	”
”	C-4544_4545, C-4553_4554, C-4556_4557	”
”	C-4559_4560, D-6_7, E-12_13	”
”	E-37_38, E-43_44, E-74_75	”
”	E-102_103	”
7st2	3-6_7, 3-53_54, 3-115_116	2.9
”	3-148_149, 3-225_226, 3-280_281	”
”	3-297_298, 3-305_306, 3-308_309	”
”	3-391_392, 3-450_451, 3-518_519	”
”	3-523_524, 3-528_529, 3-535_536	”
”	3-558_559, 3-559_560, 3-560_561	”
”	3-561_562, 3-563_564, 3-614_615	”
”	3-641_642, 3-687_688, 3-719_720	”
”	3-720_721, 3-732_733, 3-733_734	”
”	3-752_753, 3-753_754, 3-780_781	”
”	3-817_818, 3-818_819, 3-831_832	”
”	3-863_864, 3-883_884, 3-888_889	”
”	3-901_902, 3-960_961, 3-982_983	”
”	3-1079_1080, 3-1080_1081, 3-1098_1099	”
”	3-1181_1182, 3-1198_1199, 3-1281_1282	”
”	3-1289_1290, 3-1330_1331, 3-1394_1395	”
”	3-1398_1399, 3-1428_1429, 3-1431_1432	”
”	3-1447_1448, 3-1486_1487, 3-1498_1499	”
”	3-1501_1502, 3-1506_1507, 3-1518_1519	”
”	2-3_4, 2-11_12, 2-12_13	”
”	2-14_15, 2-23_24, 2-25_26	”
”	2-30_31, 2-39_40, 2-43_44	”
”	2-107_108	”
7xny	A-14_15, A-68_69, A-2463_2464	2.5
”	A-3929_3930, C-22_23, C-47_48	”
8eiu	A-12_13, A-163_164, A-194_195	2.24
”	A-200_201, A-342_343, A-344_345	”
”	A-345_346, A-508_509, A-578_579	”
”	A-801_802, A-935_936, A-1156_1157	”
”	b-8_9, b-16_17, b-104_105	”

Table B: LD data set from Section 2.1 in the main text. The PDB ID, the name of the chain and residue numbers in the suite and the resolution of the respective measurement are listed in alphabetical order.

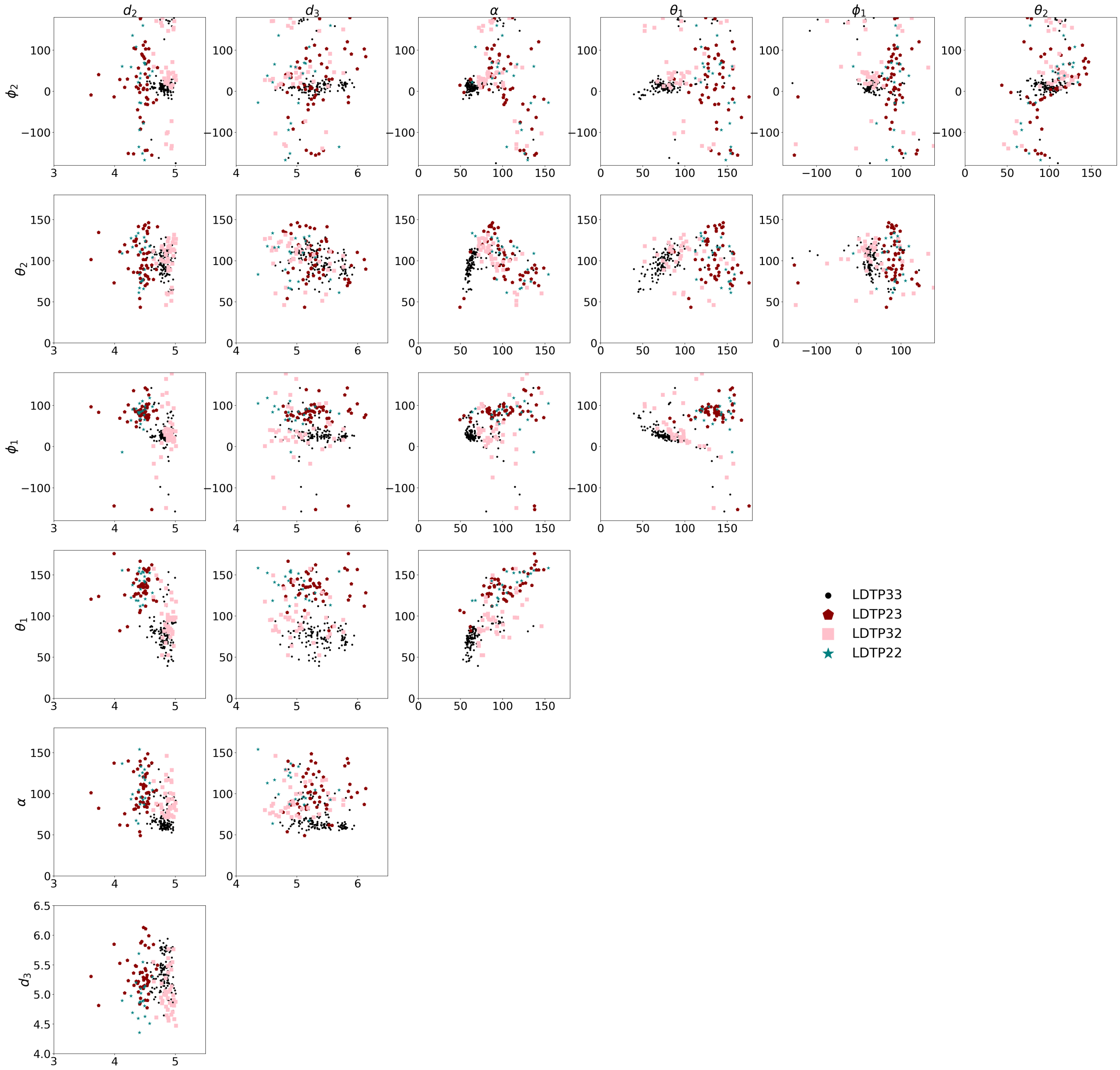

Fig C: The low detail representation of the LDT data set, resolved in pucker-pair (introduced in Section 2.1 in the main text), represented by scatter plots of all combinations of parameters (described in Section 2.3.1 in the main text).

B.2 8b0x test data

Table C lists the suites contained in the 8b0x test data set (introduced in Section 2.1). In Fig D the four pucker-pair sets of 8b0x are plotted.

PDB	suite name (chain-residues)	res
7st2	1-29_30, 1-45_46, 1-57_58	2.9
"	1-83_84, 1-95_96, 1-99_100	"
"	1-109_110, 1-148_149, 1-198_199	"
"	1-203_204, 1-218_219, 1-219_220	"
"	1-227_228, 1-228_229, 1-229_230	"
"	1-240_241, 1-251_252, 1-254_255	"
"	1-264_265, 1-265_266, 1-293_294	"
"	1-321_322, 1-322_323, 1-333_334	"
"	1-370_371, 1-371_372, 1-382_383	"
"	1-394_395, 1-395_396, 1-402_403	"
"	1-423_424, 1-442_443, 1-464_465	"
"	1-466_467, 1-474_475, 1-476_477	"
"	1-499_500, 1-500_501, 1-516_517	"
"	1-528_529, 1-561_562, 1-567_568	"
"	1-621_622, 1-633_634, 1-669_670	"
"	1-684_685, 1-694_695, 1-788_789	"
"	1-799_800, 1-818_819, 1-829_830	"
"	1-857_858, 1-865_866, 1-868_869	"
"	1-906_907, 1-940_941, 1-972_973	"
"	1-988_989, 1-998_999, 1-1002_1003	"
"	1-1004_1005, 1-1020_1021, 1-1024_1025	"
"	1-1129_1130, 1-1130_1131, 1-1138_1139	"
"	1-1154_1155, 1-1185_1186, 1-1203_1204	"
"	1-1205_1206, 1-1210_1211, 1-1211_1212	"
"	1-1235_1236, 1-1246_1247, 1-1247_1248	"
"	1-1254_1255, 1-1264_1265, 1-1274_1275	"
"	1-1344_1345, 1-1358_1359, 1-1367_1368	"
"	1-1375_1376, 1-1394_1395, 1-1418_1419	"
"	1-1426_1427, 1-1436_1437, 1-1457_1458	"
"	1-1477_1478, 1-1503_1504, 1-1521_1522	"
"	1-1553_1554, 1-1554_1555, 1-1557_1558	"
"	1-1558_1559, 1-1559_1560, 1-1626_1627	"
"	1-1633_1634, 1-1638_1639, 1-1653_1654	"
"	1-1668_1669, 1-1671_1672, 1-1693_1694	"
"	1-1697_1698, 1-1756_1757, 1-1757_1758	"
"	1-1775_1776, 1-1779_1780, 1-1785_1786	"
"	1-1818_1819, 1-1856_1857, 1-1883_1884	"
"	1-1900_1901, 1-1902_1903, 1-1926_1927	"
"	1-1935_1936, 1-1965_1966, 1-1979_1980	"
"	1-1980_1981, 1-1995_1996, 1-1996_1997	"
"	1-2021_2022, 1-2048_2049, 1-2051_2052	"
"	1-2092_2093, 1-2202_2203, 1-2242_2243	"
"	1-2249_2250, 1-2278_2279, 1-2296_2297	"
"	1-2326_2327, 1-2330_2331, 1-2344_2345	"
"	1-2349_2350, 1-2360_2361, 1-2444_2445	"
"	1-2473_2474, 1-2475_2476, 1-2479_2480	"
"	1-2553_2554, 1-2563_2564, 1-2564_2565	"
"	1-2571_2572, 1-2614_2615, 1-2645_2646	"
"	1-2715_2716, 1-2731_2732, 1-2732_2733	"
"	1-2763_2764, 1-2807_2808, 1-2808_2809	"
"	1-2817_2818, 1-2822_2823, 1-2858_2859	"
"	1-2866_2867, 1-2867_2868, 1-2871_2872	"
"	1-2886_2887, 1-2890_2891	"

PDB	suite name (chain-residues)	res
8eiu	a-44_45, a-60_61, a-130_131	2.24
"	a-537_538, a-864_865, a-1204_1205	"
"	a-1240_1241, a-1625_1626, a-1749_1750	"
"	a-1790_1791, a-1873_1874, a-1964_1965	"
"	a-2079_2080, a-2317_2318, a-2320_2321	"
"	a-2343_2344, a-2433_2434, a-2446_2447	"
"	a-2474_2475, a-2534_2535	"

Table C: LD data set from Section 2.1 in the main text. The PDB ID, the name of the chain and residue numbers in the suite and the resolution of the respective measurement are listed in alphabetical order.

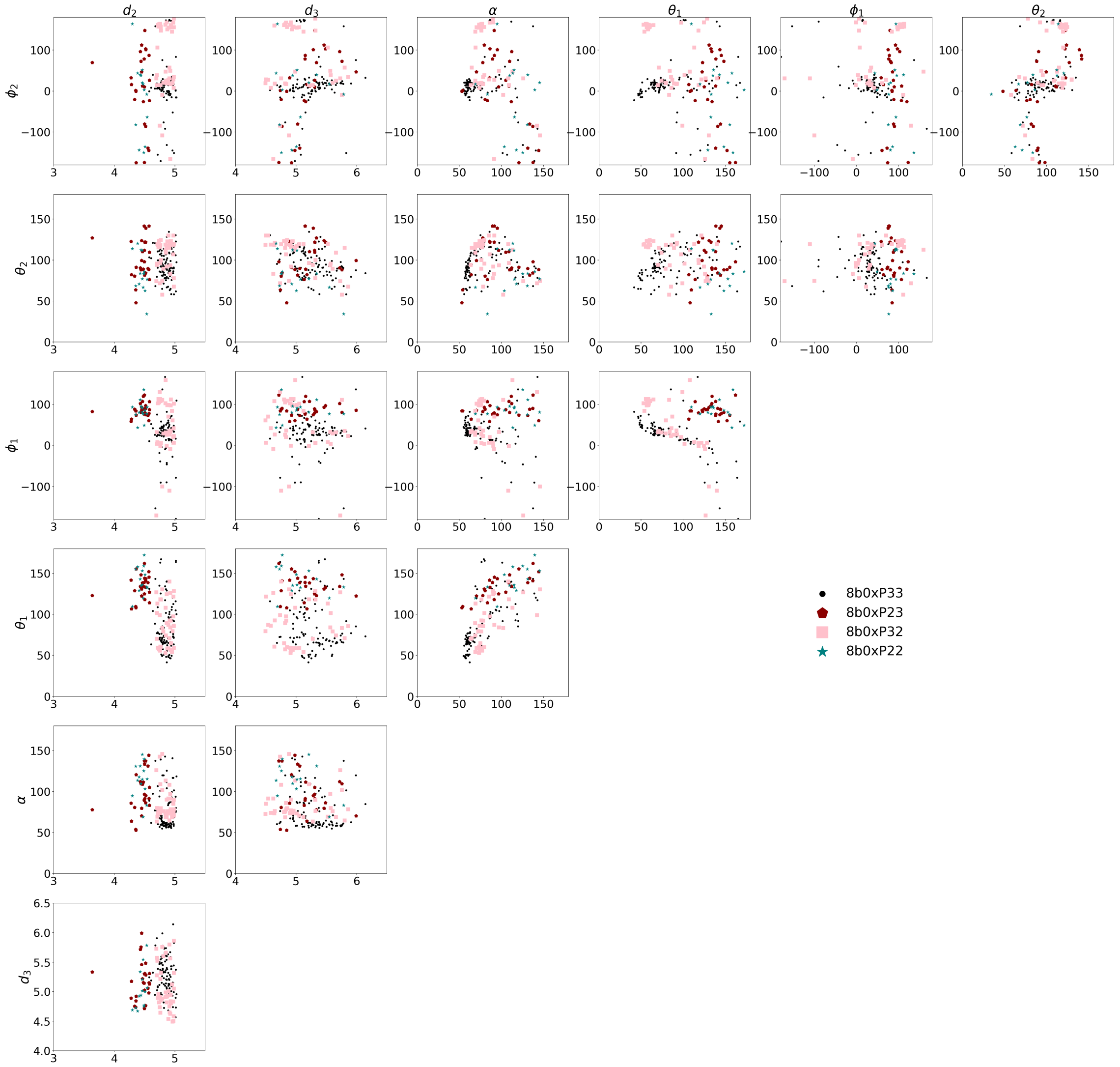

Fig D: The low detail representation of the 8b0x test data set, resolved in pucker-pair (introduced in Section 2.1 in the main text), represented by scatter plots of all combinations of parameters (described in Section 2.3.1 in the main text).

C Clustering results of HD and comparison with suitename

This section shows the clustering results of the four datasets HDP33, HDC2C3, HDP32 and HDP22 with the MINT-AGE clustering. The tuning parameters required for MINT-AGE are listed in Table 1 in the main text. The cluster results for the four different data sets are plotted in Fig E, Fig F, Fig G and Fig H. In addition, the conformer class was determined for all elements of the four pucker-pair sets with the `phenix.suitename` software. A detailed comparison between the MINT-AGE cluster results and the conformer classification with `phenix.suitename` is provided in Table D and in the confusion matrices in Fig I, Fig K, Fig J and Fig L.

HDP33

Cluster	nr elements	suite conformers
1	3214	1a (3179), 1L (14), &a (13), 1m (7), 7a (1)
2	146	1c (146)
3	43	1g (43)
4	17	9a (10), 3a (6), 7a (1)
5	11	1e (11)
6	7	1f (7)
7	4	1e (2), 1f (2)
8	3	7d (3)
9	3	5d (3)
10	3	5j (3)
11	3	1f (3)
outlier	75	

HDP23

Cluster	nr elements	suite conformers
1	57	2a (57)
2	22	0a (16), #a (4), 4a (2)
3	22	6n (19), 0i (2), 6j (1)
4	17	6g (15), 4g (2)
5	16	8d (14), 4d (2)
6	12	0a (12)
7	12	4a (12)
8	9	6d (9)
9	8	0i (8)
10	7	6j (7)
11	7	2g (6), 8d (1)
12	6	4n (6)
13	5	6d (5)
14	5	2h (5)
15	4	4g (4)
16	3	4g (2), 4a (1)
outlier	4	

HDP32

Cluster	nr elements	suite conformers
1	145	1b (121), 1[(24)
2	23	5z (23)
3	19	3b (19)
4	13	7r (13)
5	10	1[(6), 1b (3), !! (1)
6	9	1z (9)
7	8	1t (8)
8	7	1o (7)
9	7	7p (7)
10	5	7p (5)
11	4	5p (4)
12	4	5p (4)
13	4	7p (4)
14	3	7p (3)
15	3	5q (3)
outlier	1	

HDP22

Cluster	nr elements	suite conformers
1	41	2[(41)
2	33	6p (32), !! (1)
3	17	4p (17)
4	15	4b (15)
5	8	2z (8)
6	6	2u (3), 2o (2), 2[(1)
7	6	4b (4), 0b (2)
8	4	0b (4)
9	4	0b (4)
10	3	4b (3)
outlier	1	

Table D: Left and middle column of the tables: MINT-AGE cluster numbers and outliers (left column) with size (middle column) for the different data HDP33, HDP32, HDP23 and HDP22 (introduced in Section 2.1 in the main text). Right column: the corresponding number of elements in the suitename two-digit conformer classes (the name of the class is a number and a letter or a bracket).

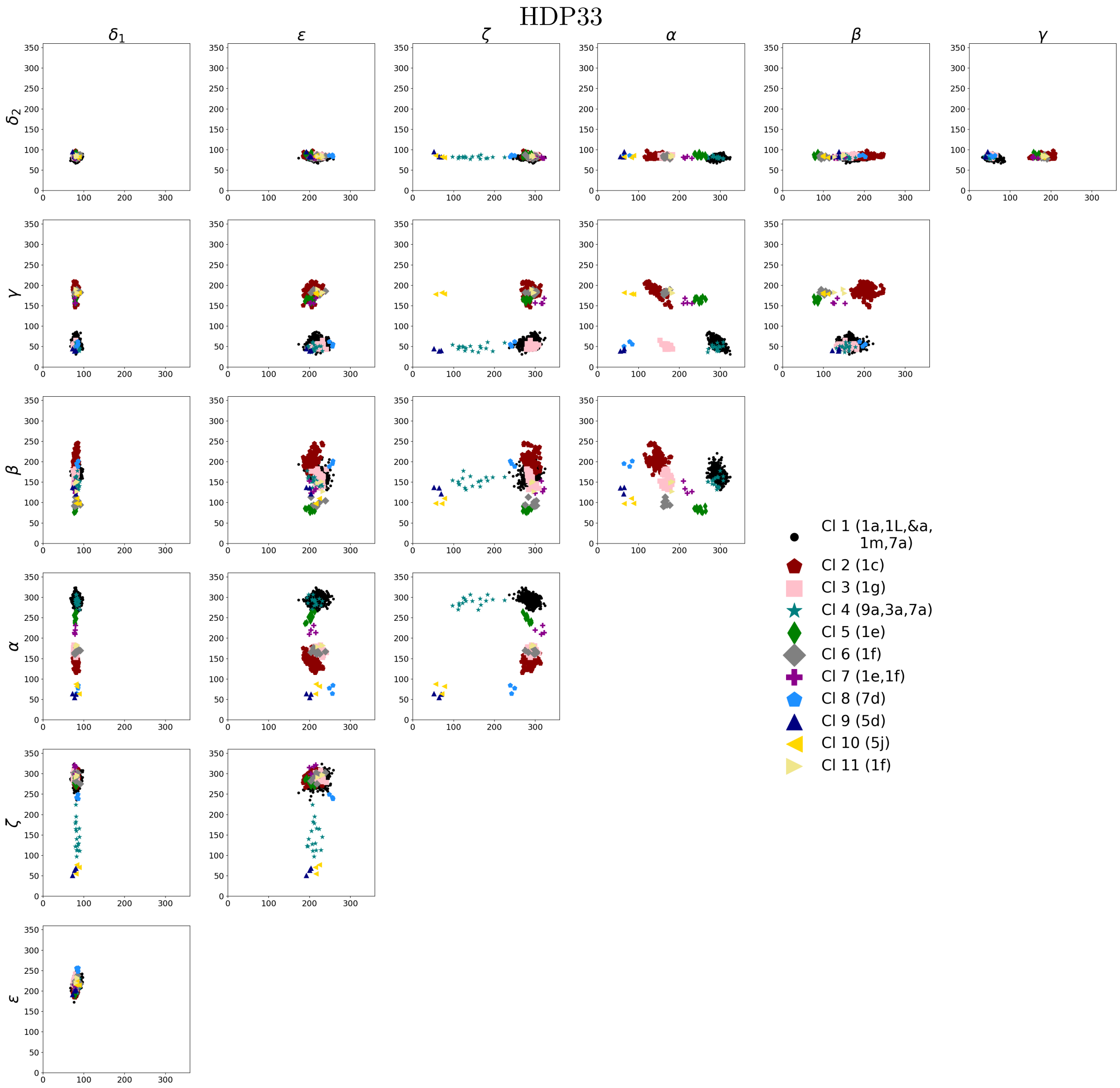

Fig E: The MINT-AGE clusters (see Section 2.2 in the main text) from the data set HDP33 (introduced in Section 2.1 in the main text) represented by scatter plots of all two-dimensional dihedral angle pairs (given in degrees). See Table D for a detailed overview of the clusters and see Fig I for a detailed comparison with the conformer classification obtained by the `phenix.suitename` software.

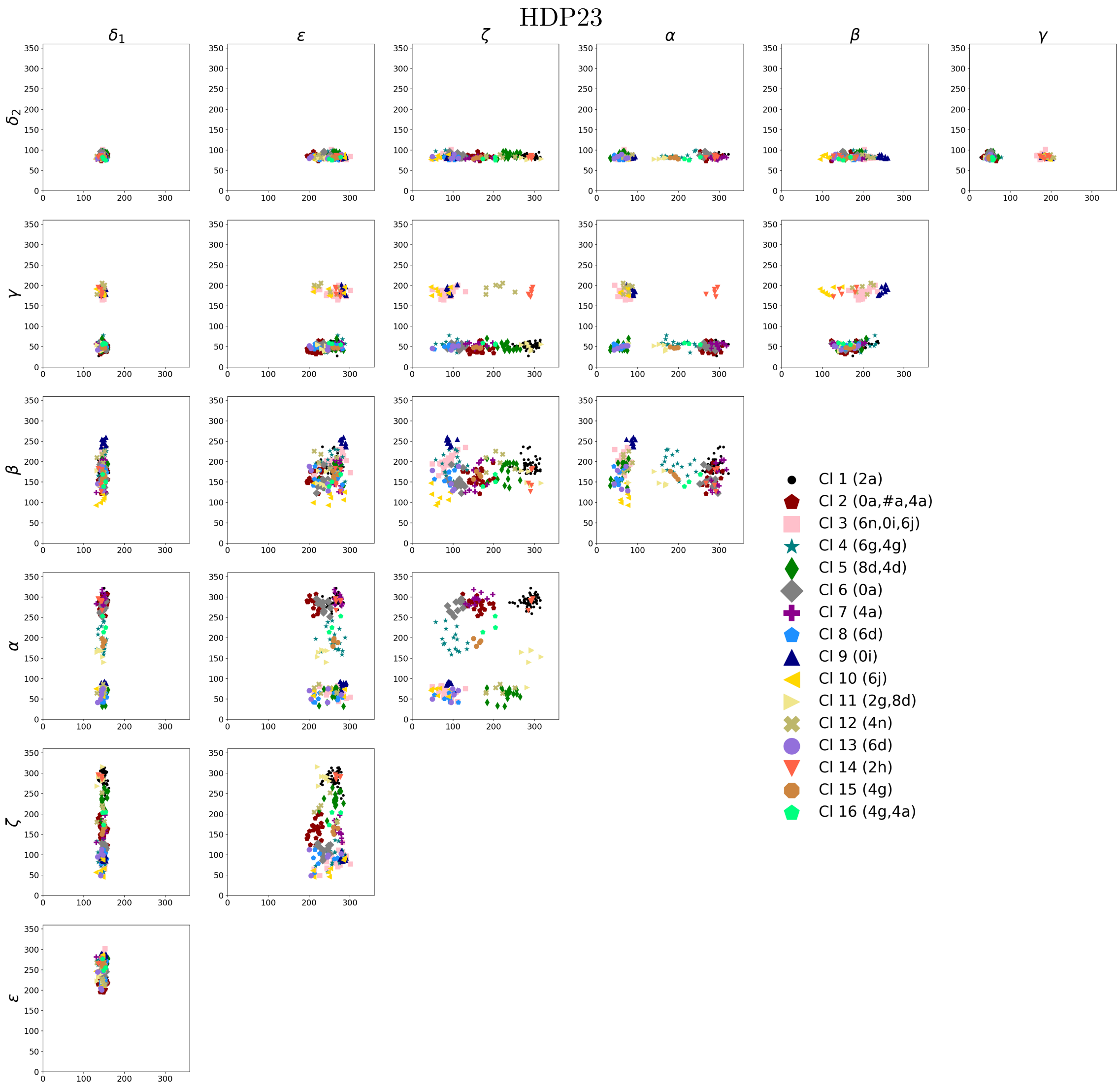

Fig F: The MINT-AGE clusters (see Section 2.2 in the main text) from the data set HDP23 (introduced in Section 2.1 in the main text) represented by scatter plots of all two-dimensional dihedral angle pairs (given in degrees). See Table D for a detailed overview of the clusters and see Fig K for a detailed comparison with the conformer classification obtained by the `phenix.suitename` software.

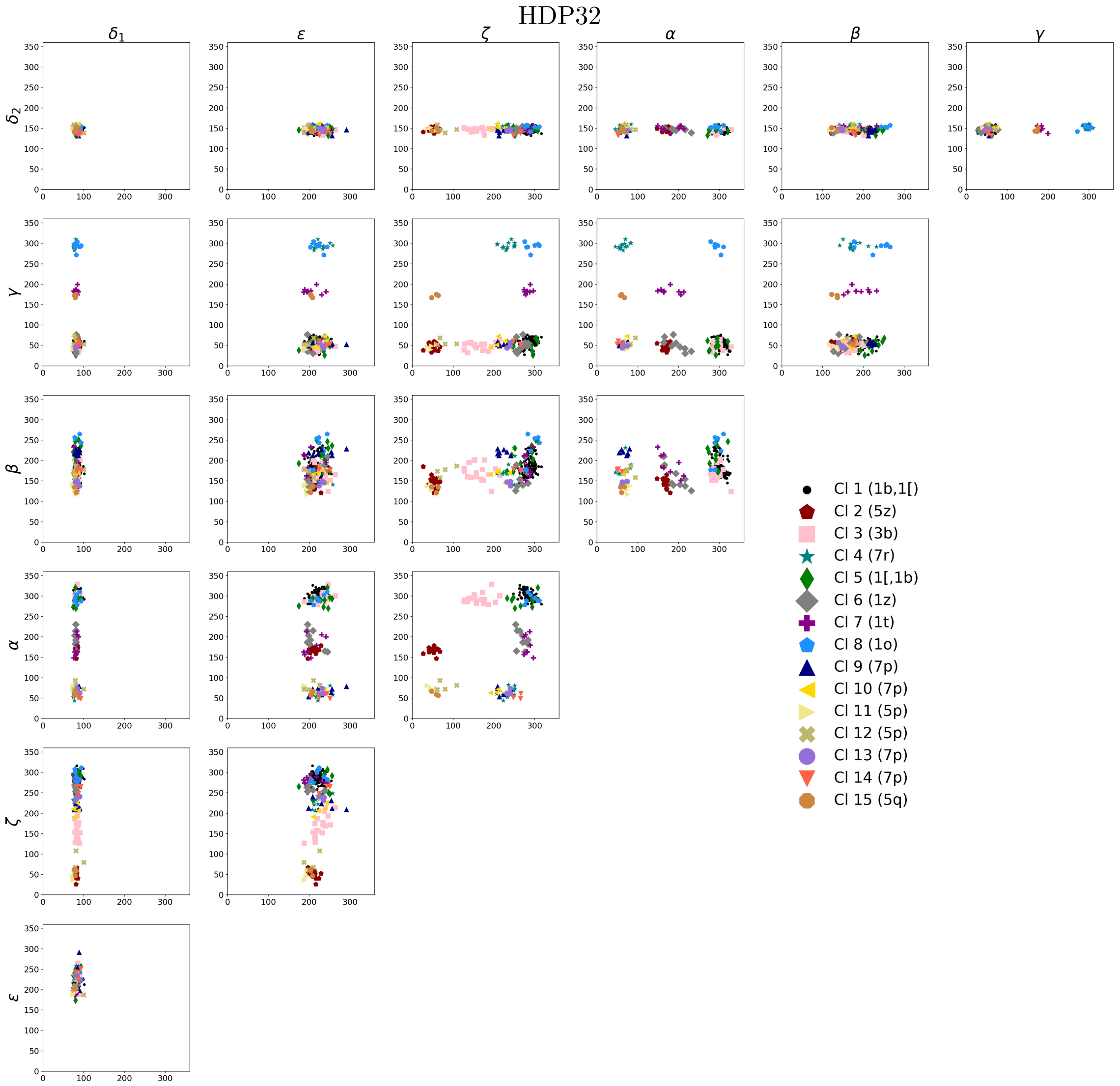

Fig G: The MINT-AGE clusters (see Section 2.2 in the main text) from the data set HDP32 (introduced in Section 2.1 in the main text) represented by scatter plots of all two-dimensional dihedral angle pairs (given in degrees). See Table D for a detailed overview of the clusters and see Fig J for a detailed comparison with the conformer classification obtained by the `phenix.suitename` software.

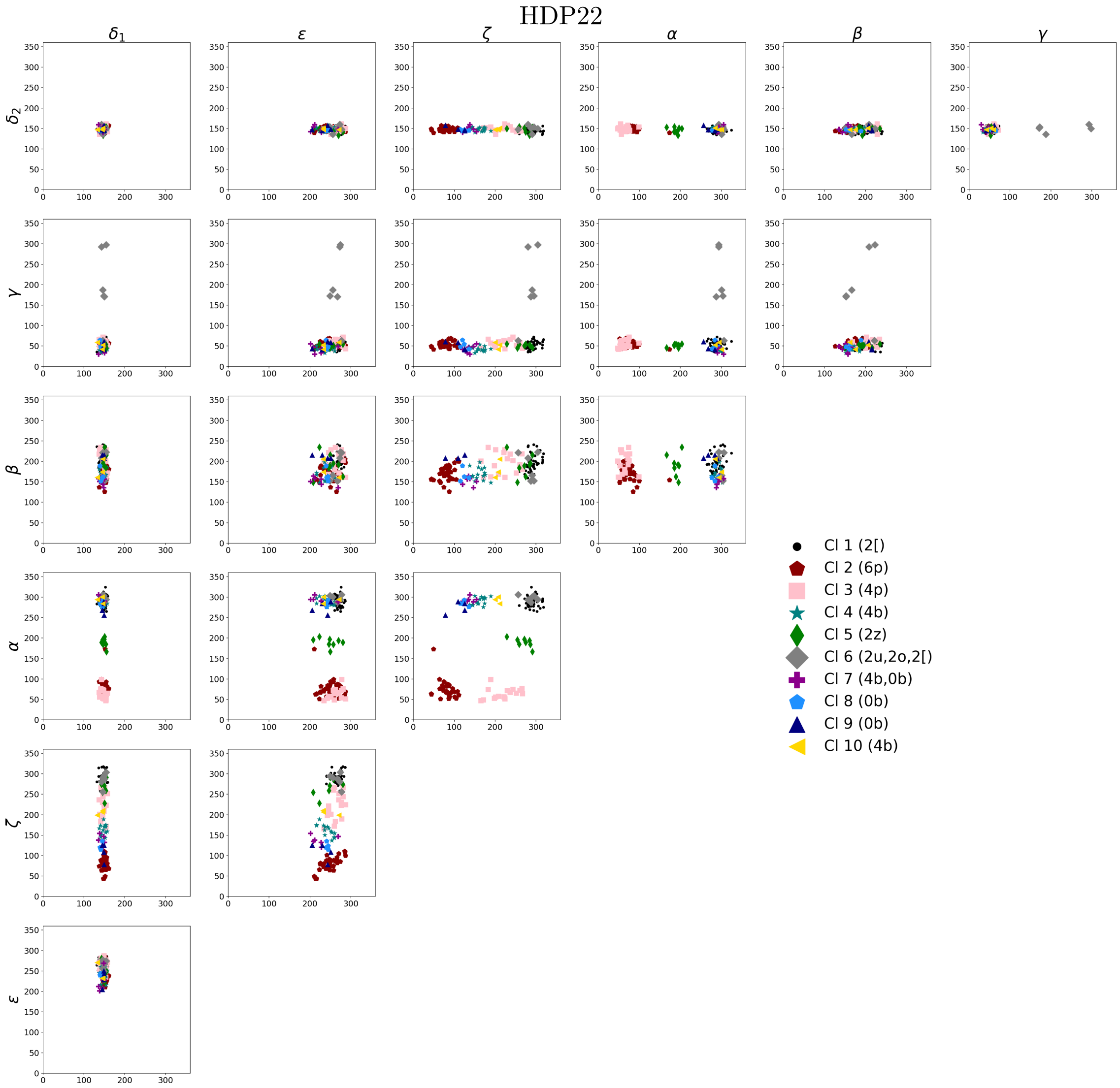

Fig H: The MINT-AGE clusters (see Section 2.2 in the main text) from the data set HDP22 (introduced in Section 2.1 in the main text) represented by scatter plots of all two-dimensional dihedral angle pairs (given in degrees). See Table D for a detailed overview of the clusters and see Fig K for a detailed comparison with the conformer classification obtained by the `phenix.suitename` software.

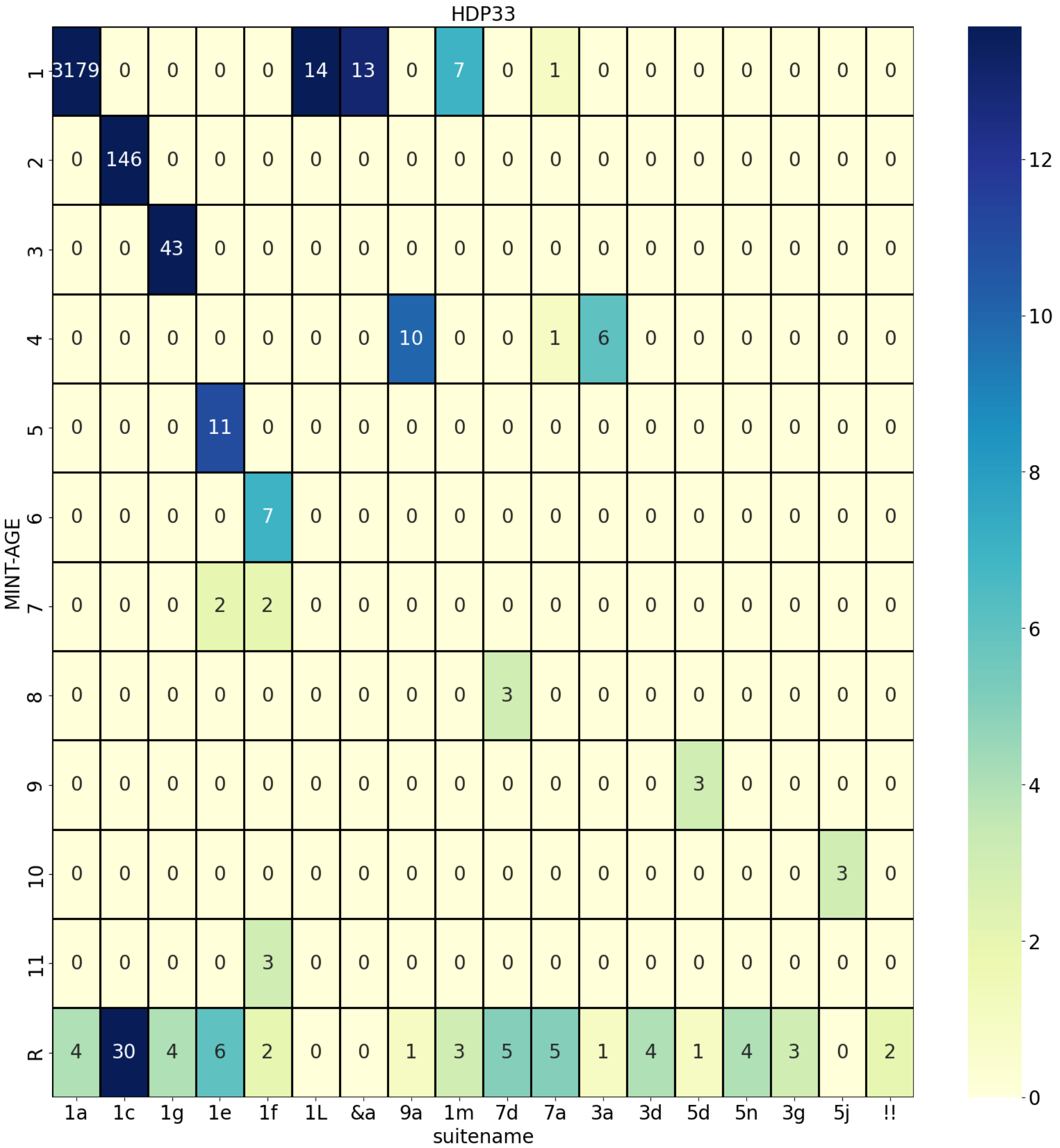

Fig I: Confusion matrix comparing the MINT-AGE cluster results with suite conformers for HDP33 training data. Every line in the matrix corresponds to a cluster and every column corresponds to a suite conformer. The last line (labeled R for rest) summarizes suites that were not assigned to any cluster by the MINT-AGE algorithm. One can see that most conformers are assigned to a single cluster with overwhelming majority. However, some clusters pool together several conformers.

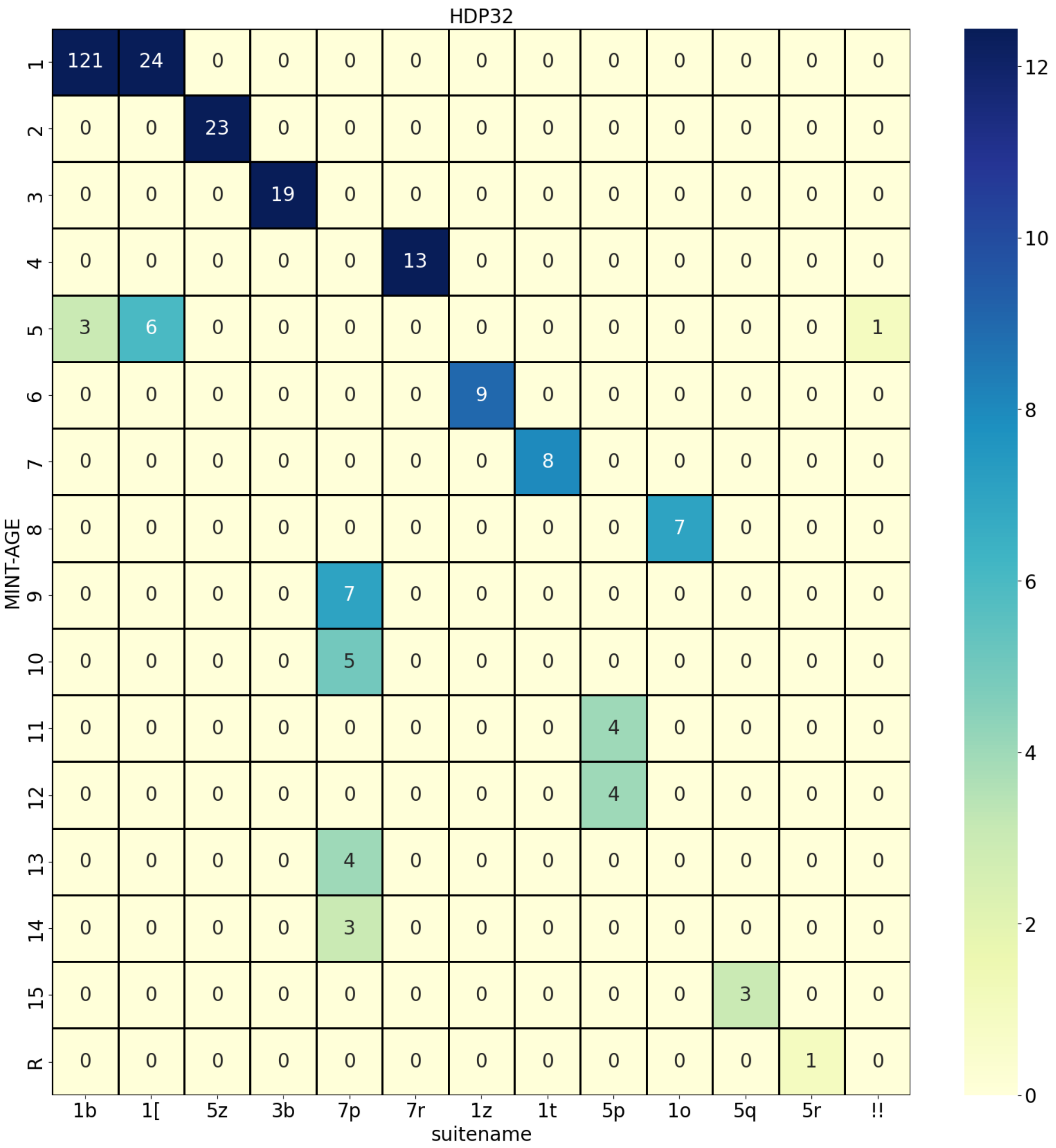

Fig J: Confusion matrix comparing the MINT-AGE cluster results with suite conformers for HDP32 training data. Every line in the matrix corresponds to a cluster and every column corresponds to a suite conformer. The last line summarizes outliers that were not assigned to any cluster by the MINT-AGE algorithm. Most clusters only comprise a single conformer, except for clusters 1 and 5 which contain both 1b and 1[conformers. Additionally, the 7p and 5p conformers are split up into several cluster.

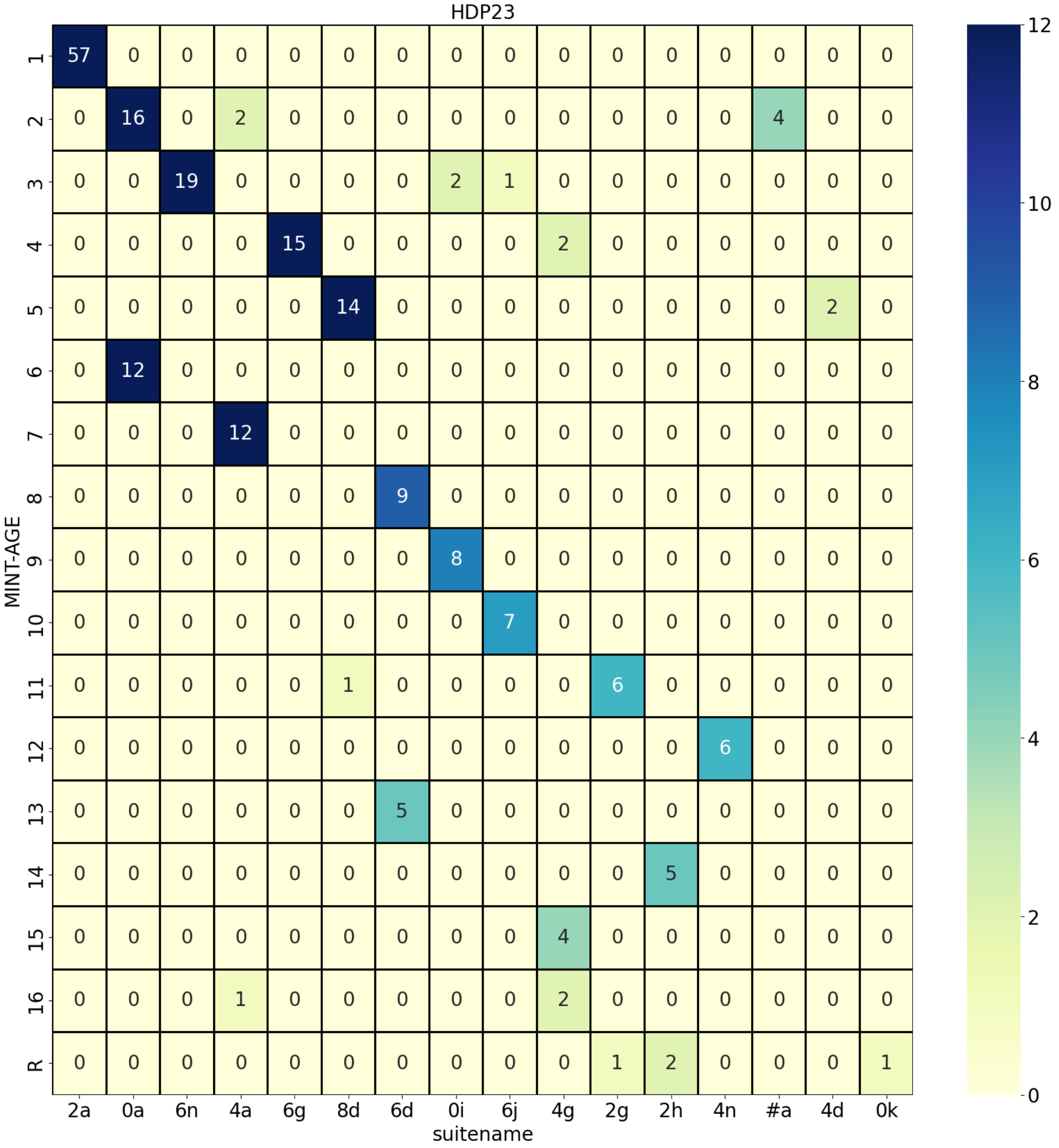

Fig K: Confusion matrix comparing the MINT-AGE cluster results with suite conformers for HDP23 training data. Every line in the matrix corresponds to a cluster and every column corresponds to a suite conformer. The last line summarizes outliers that were not assigned to any cluster by the MINT-AGE algorithm. Most clusters only comprise a single conformer, except for cluster 2 containing both 0a and #a conformers. Additionally, the 0a and 6d conformers are split up into several cluster.

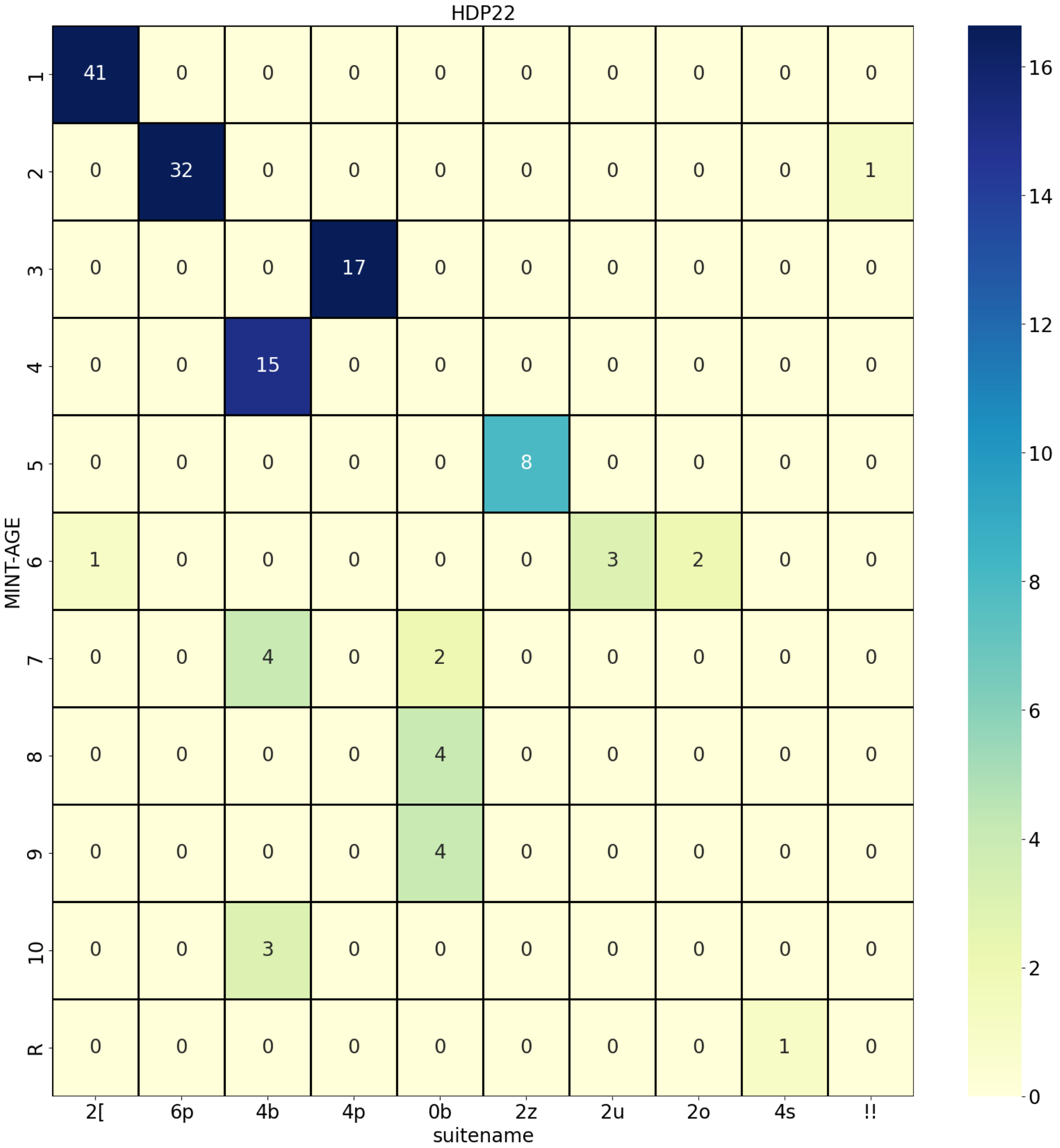

Fig L: Confusion matrix comparing the MINT-AGE cluster results with suite conformers for HDP22 training data. Every line in the matrix corresponds to a cluster and every column corresponds to a suite conformer. The last line summarizes outliers that were not assigned to any cluster by the MINT-AGE algorithm. While bigger clusters correspond pretty well with abundant conformers, the picture is less clear cut for smaller clusters and rare conformers.

D Low detail (LD) parameter space

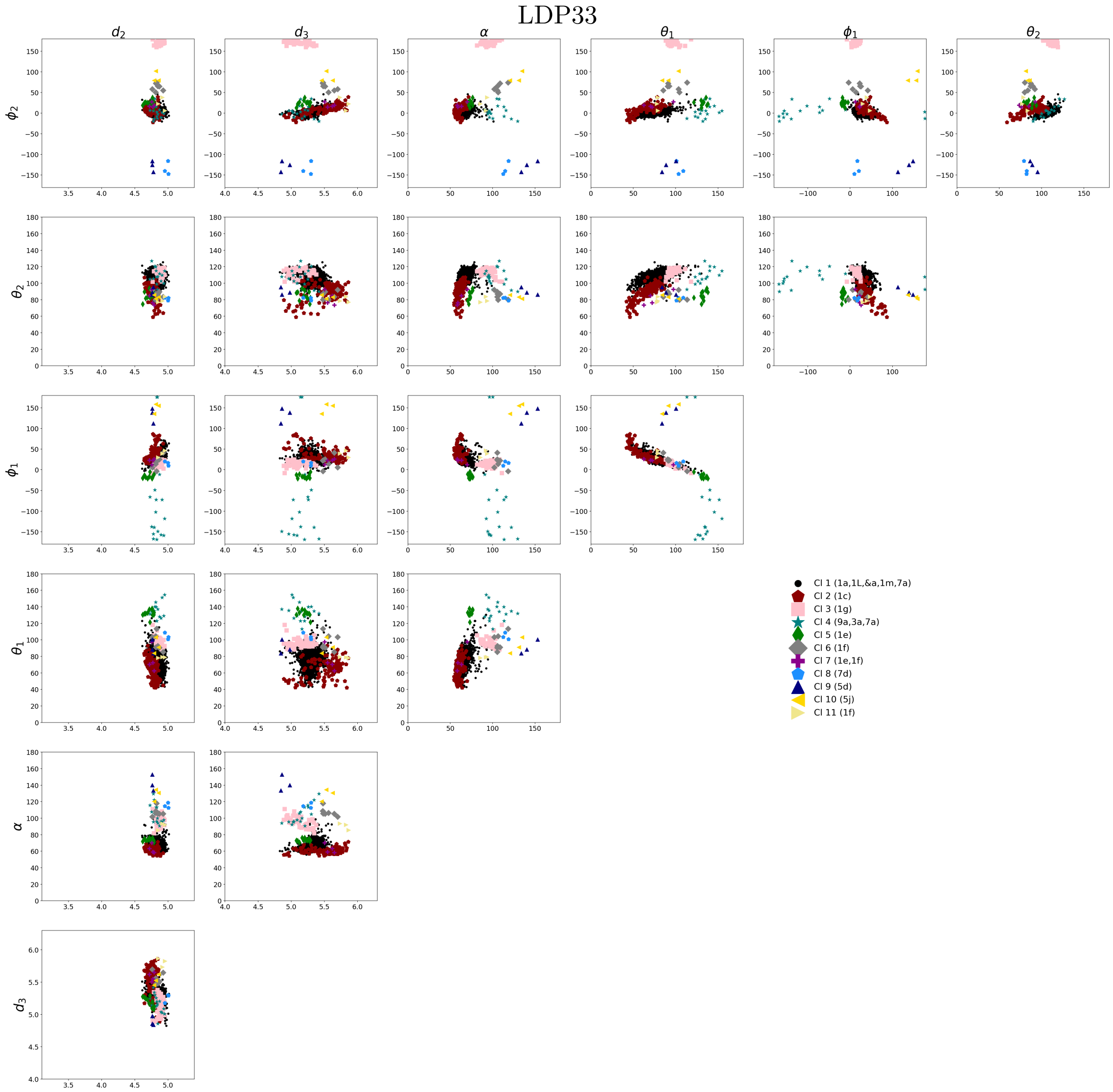

Fig M: Scatter plot of LDP33 data in low detail parameter space introduced in Section 2.3.1 of the main text.

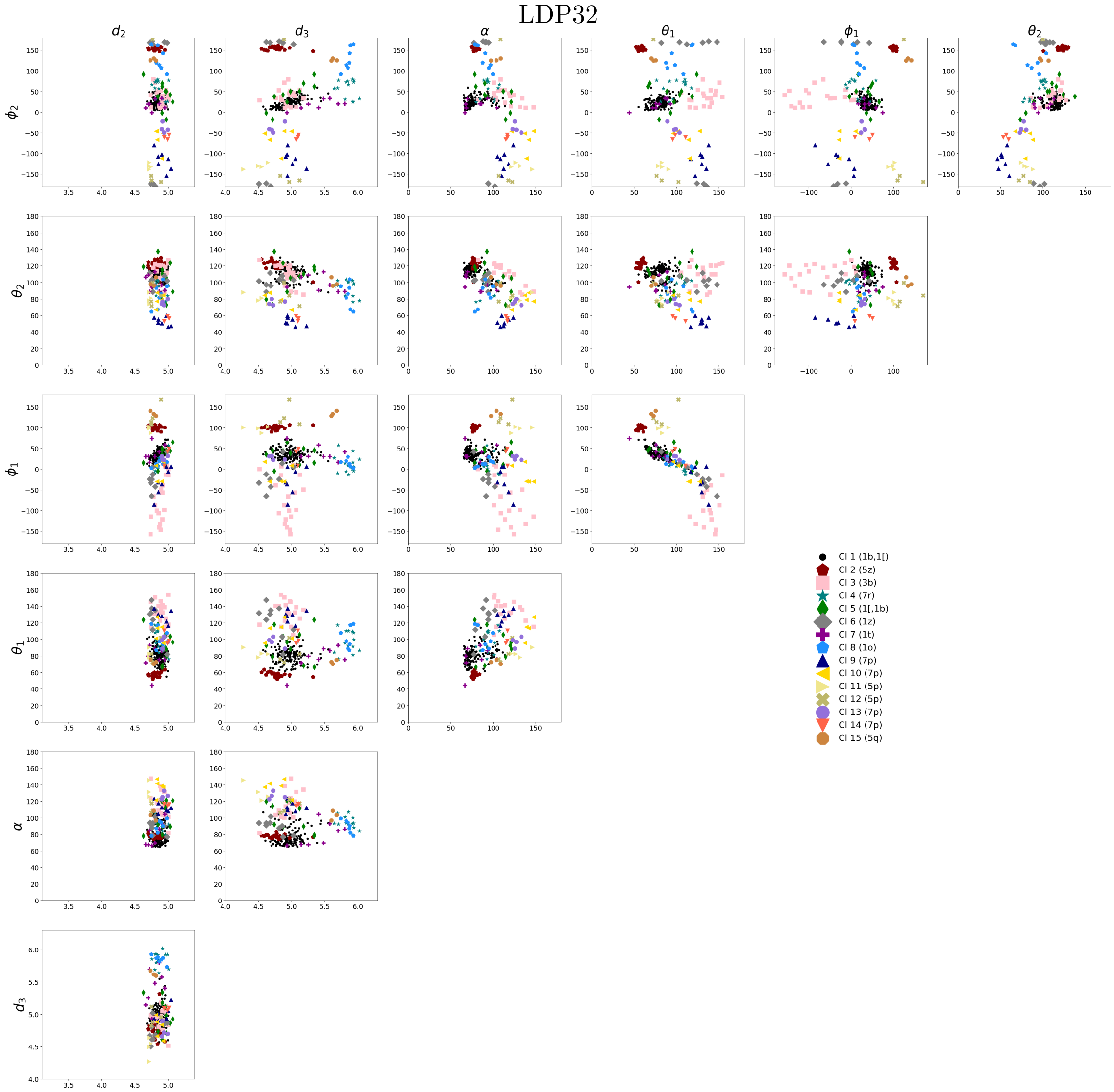

Fig N: Scatter plot of LDP32 data in low detail parameter space introduced in Section 2.3.1 of the main text.

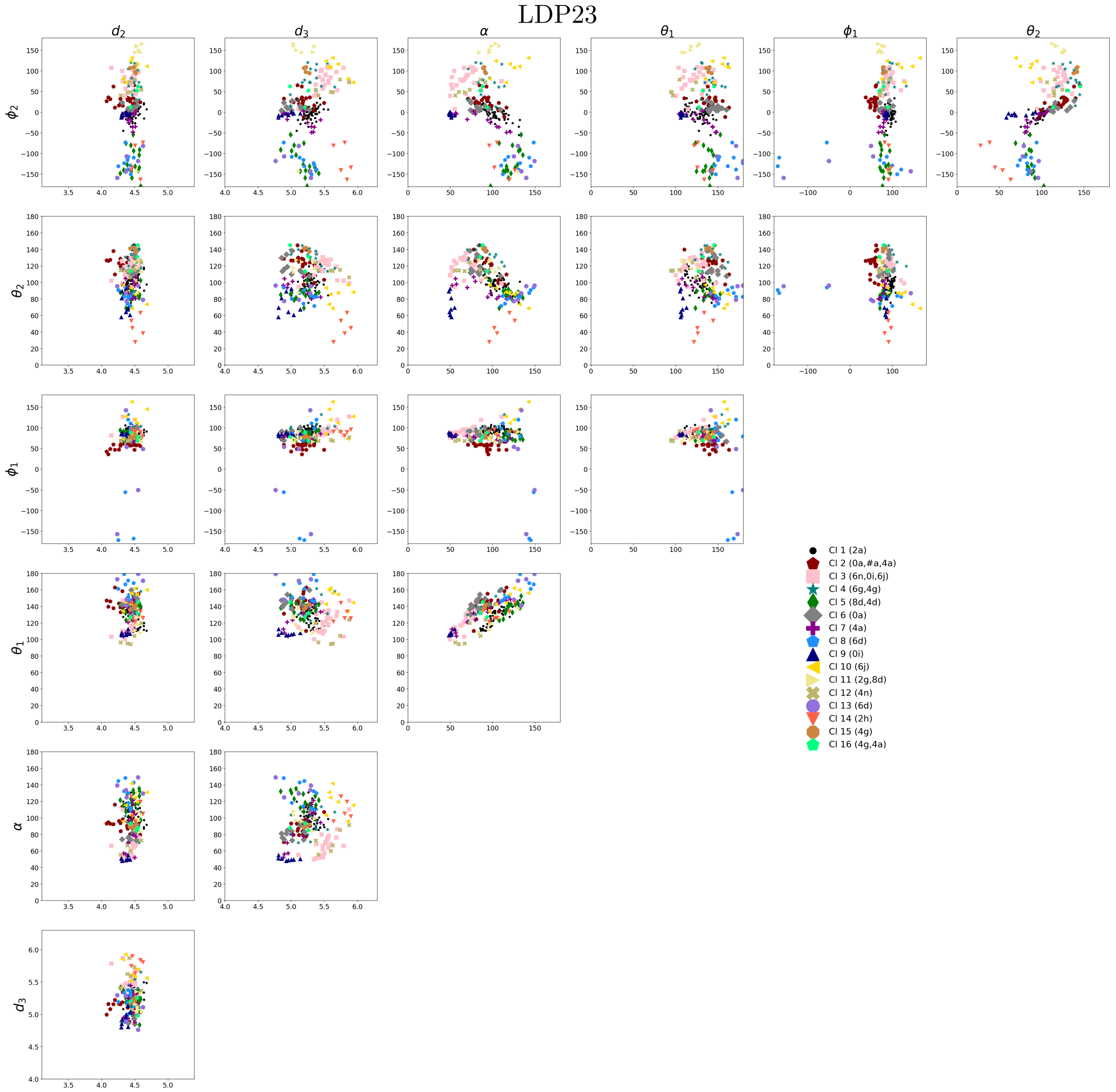

Fig O: Scatter plot of LDP23 data in low detail parameter space introduced in Section 2.3.1 of the main text.

LDP22

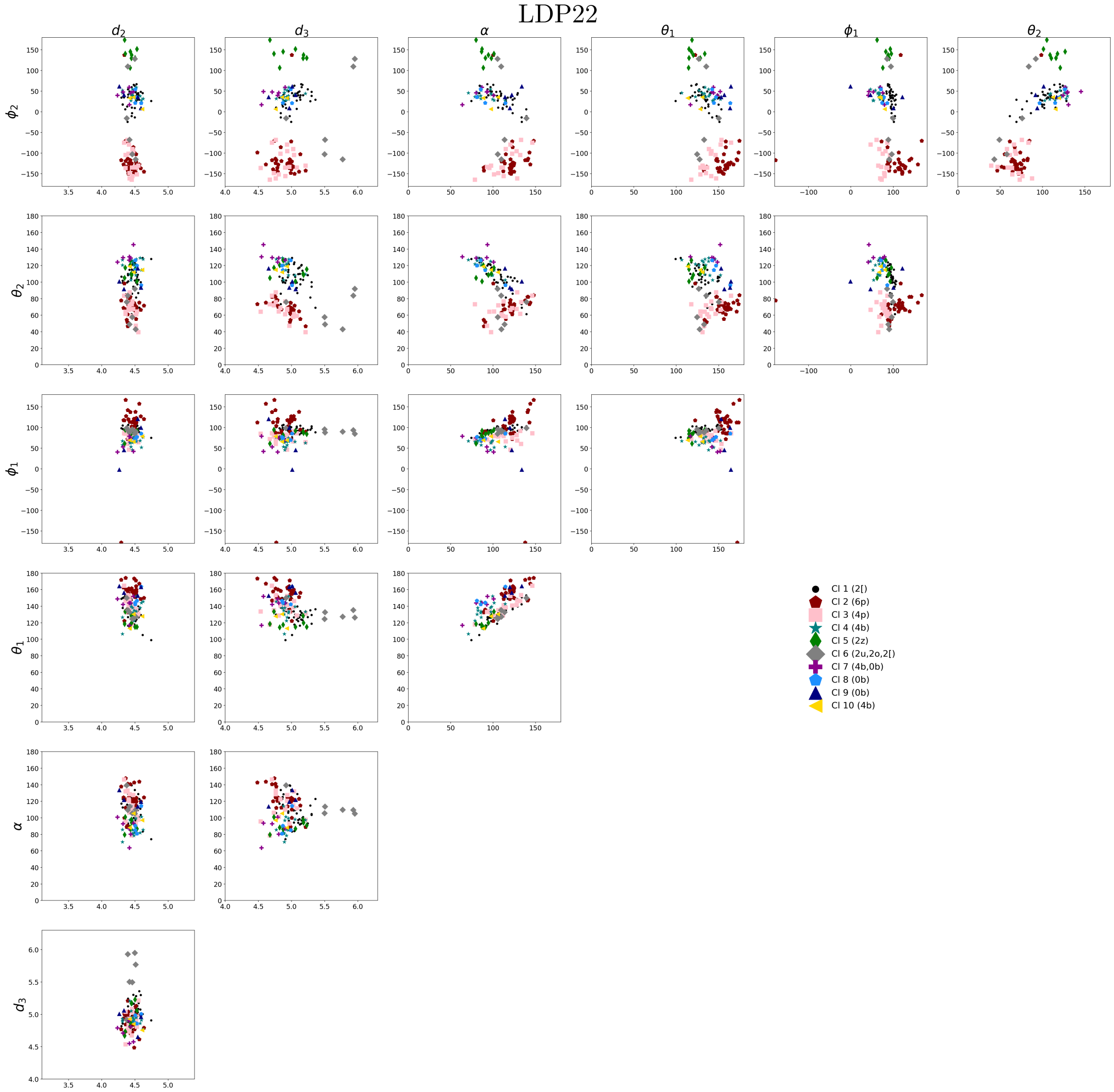

Fig P: Scatter plot of LDP22 data in low detail parameter space introduced in Section 2.3.1 of the main text.

E Results RNAprecis

E.1 Results for the test data set LDTP33

LDTP33: matches

name	p. answer	RNAprecis	p_{post}
2eev_A-79_A-80	1a	**1**=(1a,1L,&a,1m,7a)	0.55
5fk1_A-16_A-17	1a	**1**=(1a,1L,&a,1m,7a)	0.57
3bsn_T-5_T-6	1a	**1**=(1a,1L,&a,1m,7a)	0.68
7f5s_LI-55_LI-56	1e	**7**=(1e,1f)	0.7
7f5s_LI-2737_LI-2738	1a	**1**=(1a,1L,&a,1m,7a)	0.86
3oij_C-2_C-3	1c	**2**=(1c)	0.89
7f5s_LJ-6_LJ-7	1f	**7**=(1e,1f)	0.9
5dhb_C-1_C-2	1a	**1**=(1a,1L,&a,1m,7a)	0.93
7st2_3-1289_3-1290	1a	**1**=(1a,1L,&a,1m,7a)	0.96
7f5s_LI-940_LI-941	1c	**2**=(1c)	0.96
2qbz_X-144_X-145	1a	**1**=(1a,1L,&a,1m,7a)	0.97
8eiu_A-935_A-936	1c	**2**=(1c)	0.97
7st2_3-53_3-54	1c	**2**=(1c)	0.98
7st2_3-1486_3-1487	1a	**1**=(1a,1L,&a,1m,7a)	0.99
7f5s_LI-1888_LI-1889	1a	**1**=(1a,1L,&a,1m,7a)	0.99
8eiu_A-200_A-201	1a	**1**=(1a,1L,&a,1m,7a)	0.99
4w5t_B-12_B-13	1a	**1**=(1a,1L,&a,1m,7a)	1.0
7st2_3-308_3-309	1a	**1**=(1a,1L,&a,1m,7a)	1.0
7f5s_LI-16_LI-17	1c	**2**=(1c)	1.0
7f5s_LI-2401_LI-2402	1c	**2**=(1c)	1.0
2qbz_X-125_X-126	3a	**4**=(9a,3a,7a)	1.0
8eiu_A-194_A-195	1a	**1**=(1a,1L,&a,1m,7a)	1.0
7xny_SX-14_SX-15	1m	**1**=(1a,1L,&a,1m,7a)	1.0
3t5q_L-4_L-5	1c	**2**=(1c)	1.0
7st2_3-1428_3-1429	1c	**2**=(1c)	1.0
8eiu_b-8_b-9	1c	**2**=(1c)	1.0
7f5s_LI-3869_LI-3870	1c	**2**=(1c)	1.0
7f5s_LI-4390_LI-4391	1c	**2**=(1c)	1.0
6u8k_C-160_C-161	1a	**1**=(1a,1L,&a,1m,7a)	1.0
7f5s_LI-976_LI-977	1c	**2**=(1c)	1.0
7f5s_LL-12_LL-13	1c	**2**=(1c)	1.0
6d8f_H-10_H-11	1a	**1**=(1a,1L,&a,1m,7a)	1.0
7st2_3-614_3-615	1c	**2**=(1c)	1.0
7st2_3-391_3-392	1c	**2**=(1c)	1.0
6q57_A-82_A-83	1a	**1**=(1a,1L,&a,1m,7a)	1.0
7eem_A-2652_A-2653	1a	**1**=(1a,1L,&a,1m,7a)	1.0
4u3p_B-6_B-7	1c	**2**=(1c)	1.0
2eet_A-17_A-18	1c	**2**=(1c)	1.0
4lvv_A-81_A-82	1c	**2**=(1c)	1.0
7f5s_LI-4224_LI-4225	1c	**2**=(1c)	1.0
7eem_A-2660_A-2661	1L	**1**=(1a,1L,&a,1m,7a)	1.0
3gx5_A-15_A-16	1a	**1**=(1a,1L,&a,1m,7a)	1.0
7xny_SX-68_SX-69	1c	**2**=(1c)	1.0
8eiu_A-578_A-579	1c	**2**=(1c)	1.0
3gx5_A-29_A-30	1c	**2**=(1c)	1.0
2qbz_X-170_X-171	1a	**1**=(1a,1L,&a,1m,7a)	1.0
8eiu_A-801_A-802	1c	**2**=(1c)	1.0
7e9i_A-25_A-26	1c	**2**=(1c)	1.0
7st2_3-780_3-781	1f	**6**=(1f)	1.0
6q57_A-34_A-35	1c	**2**=(1c)	1.0
2eet_A-19_A-20	1a	**1**=(1a,1L,&a,1m,7a)	1.0
5fk1_A-90_A-91	1a	**1**=(1a,1L,&a,1m,7a)	1.0
7f5s_LI-2815_LI-2816	1a	**1**=(1a,1L,&a,1m,7a)	1.0
3bsn_T-6_T-7	1a	**1**=(1a,1L,&a,1m,7a)	1.0
7st2_3-831_3-832	1c	**2**=(1c)	1.0
7f5s_LI-1620_LI-1621	1a	**1**=(1a,1L,&a,1m,7a)	1.0
7f5s_LI-3925_LI-3926	1c	**2**=(1c)	1.0
4fnj_A-4_A-5	1c	**2**=(1c)	1.0
7f5s_LL-43_LL-44	1a	**1**=(1a,1L,&a,1m,7a)	1.0
5fk1_A-56_A-57	1c	**2**=(1c)	1.0
6d8f_H-16_H-17	1c	**2**=(1c)	1.0
5dhb_C-2_C-3	1a	**1**=(1a,1L,&a,1m,7a)	1.0
6h9i_E-8_E-9	1c	**2**=(1c)	1.0
8eiu_b-16_b-17	1c	**2**=(1c)	1.0
6q57_A-87_A-88	1a	**1**=(1a,1L,&a,1m,7a)	1.0
2qbz_X-35_X-36	1a	**1**=(1a,1L,&a,1m,7a)	1.0

LDTP33: matches

name	p. answer	RNAprecis	p_{post}
3umy_B-2181_B-2182	1c	**2**=(1c)	1.0
2qbz_X-171_X-172	1a	**1**=(1a,1L,&a,1m,7a)	1.0
4yn6_A-9_A-10	1a	**1**=(1a,1L,&a,1m,7a)	1.0
2eev_A-26_A-27	1a	**1**=(1a,1L,&a,1m,7a)	1.0
7st2_3-225_3-226	1c	**2**=(1c)	1.0
2qbz_X-113_X-114	1c	**2**=(1c)	1.0
3gx5_A-52_A-53	1a	**1**=(1a,1L,&a,1m,7a)	1.0
5fk1_A-50_A-51	1g	**3**=(1g)	1.0
7st2_3-297_3-298	1g	**3**=(1g)	1.0
6y0t_A-2659_A-2660	1g	**3**=(1g)	1.0
7f5s_LI-4544_LI-4545	1c	**2**=(1c)	1.0
7f5s_LI-4498_LI-4499	1g	**3**=(1g)	1.0
5npm_B-3_B-4	1c	**2**=(1c)	1.0
7f5s_LI-1393_LI-1394	1c	**2**=(1c)	1.0
5neo_A-7_A-8	5d	**9**=(5d)	1.0
7st2_3-901_3-902	1c	**2**=(1c)	1.0
7st2_3-1080_3-1081	1c	**2**=(1c)	1.0
7st2_3-863_3-864	1g	**3**=(1g)	1.0
4yn6_A-2_A-3	1g	**3**=(1g)	1.0
4xw7_A-24_A-25	1c	**2**=(1c)	1.0
7st2_3-523_3-524	1g	**3**=(1g)	1.0
3gx5_A-23_A-24	5d	**9**=(5d)	1.0
7f5s_LL-74_LL-75	1g	**3**=(1g)	1.0
7f5s_LI-1524_LI-1525	1c	**2**=(1c)	1.0
6u8k_C-201_C-219	1c	**2**=(1c)	1.0

Table E: Suites for which the RNAprecis predicted cluster matches the sequence related conformer (blue in the plots) sorted by posterior probability.

LDTP33: pucker mismatches

name	p. answer	p_1	p_2	RNAprecis	p_{post}
3gx5_A-68_A-69	2a	2.91	4.66	4=(9a,3a,7a)	0.7
7e9i_A-31_A-32	0a	4.41	4.46	1=(1a,1L,&a,1m,7a)	0.86
3q51_A-30_A-31	1b	4.48	4.44	1=(1a,1L,&a,1m,7a)	1.0
4z4g_D-7_D-8	1b	4.6	4.64	1=(1a,1L,&a,1m,7a)	1.0
7st2_3-1281_3-1282	2a	3.83	4.61	4=(9a,3a,7a)	1.0
7st2_3-1330_3-1331	1b	4.63	3.47	2=(1c)	1.0
7st2_3-1447_3-1448	2a	3.95	4.54	4=(9a,3a,7a)	1.0
7st2_2-107_2-108	1[	4.56	3.85	2=(1c)	1.0

LDTP33: conformer mismatches

name	p. answer	RNAprecis	p_{post}	first match	p_{post}
7f5s_LI-3884_LI-3885	1c	1=(1a,1L,&a,1m,7a)	0.58	2=(1c)	0.42
8eiu_A-163_A-164	1e	2=(1c)	0.67	7=(1e,1f)	0.33
5b2o_B-17_B-18	1e	2=(1c)	0.96	7=(1e,1f)	0.04
4w5t_B-8_B-9	1a	7=(1e,1f)	0.97	1=(1a,1L,&a,1m,7a)	$4 \cdot 10^{-11}$
8eiu_b-104_b-105	1a	2=(1c)	0.97	1=(1a,1L,&a,1m,7a)	0.03
7f5s_LI-4553_LI-4554	1c	1=(1a,1L,&a,1m,7a)	0.99	2=(1c)	$6 \cdot 10^{-3}$
8eiu_A-342_A-343	1a	2=(1c)	0.99	1=(1a,1L,&a,1m,7a)	$6 \cdot 10^{-3}$
7st2_2-30_2-31	1a	2=(1c)	1.0	1=(1a,1L,&a,1m,7a)	$2 \cdot 10^{-3}$
7f5s_LI-1585_LI-1586	1a	2=(1c)	1.0	1=(1a,1L,&a,1m,7a)	$1 \cdot 10^{-3}$
4yn6_B-1_B-2	1a	2=(1c)	1.0	1=(1a,1L,&a,1m,7a)	$1 \cdot 10^{-4}$
6q57_A-53_A-54	1a	2=(1c)	1.0	1=(1a,1L,&a,1m,7a)	$4 \cdot 10^{-5}$
3i5x_B-3_B-4	1a	4=(9a,3a,7a)	1.0	1=(1a,1L,&a,1m,7a)	$3 \cdot 10^{-8}$
6h9i_E-12_E-13	1a	3=(1g)	1.0	1=(1a,1L,&a,1m,7a)	$2 \cdot 10^{-206}$
7st2_3-1098_3-1099	1a	2=(1c)	1.0	1=(1a,1L,&a,1m,7a)	$7 \cdot 10^{-6}$
7xny_SX-3929_SX-3930	1a	2=(1c)	1.0	1=(1a,1L,&a,1m,7a)	$5 \cdot 10^{-6}$
7st2_3-148_3-149	1f	2=(1c)	1.0	7=(1e,1f)	$3 \cdot 10^{-6}$
6u8k_C-157_C-158	1a	2=(1c)	1.0	1=(1a,1L,&a,1m,7a)	$10 \cdot 10^{-7}$
7f5s_LI-3684_LI-3685	1a	2=(1c)	1.0	1=(1a,1L,&a,1m,7a)	$4 \cdot 10^{-7}$
4fnj_A-30_A-31	1a	2=(1c)	1.0	1=(1a,1L,&a,1m,7a)	$9 \cdot 10^{-8}$
2eet_A-44_A-45	1a	2=(1c)	1.0	1=(1a,1L,&a,1m,7a)	$8 \cdot 10^{-8}$
7st2_3-563_3-564	3g	4=(9a,3a,7a)	1.0	-	-
7st2_2-3_2-4	1a	2=(1c)	1.0	1=(1a,1L,&a,1m,7a)	$4 \cdot 10^{-49}$
4fnj_A-3_A-4	1a	2=(1c)	1.0	1=(1a,1L,&a,1m,7a)	$7 \cdot 10^{-9}$
7f5s_LI-2411_LI-2412	1a	2=(1c)	1.0	1=(1a,1L,&a,1m,7a)	$2 \cdot 10^{-9}$
7st2_3-1079_3-1080	1a	2=(1c)	1.0	1=(1a,1L,&a,1m,7a)	$1 \cdot 10^{-9}$
7st2_2-25_2-26	1a	2=(1c)	1.0	1=(1a,1L,&a,1m,7a)	$1 \cdot 10^{-9}$
7f5s_LL-102_LL-103	1a	6=(1f)	1.0	1=(1a,1L,&a,1m,7a)	$2 \cdot 10^{-19}$
7st2_3-528_3-529	1a	2=(1c)	1.0	1=(1a,1L,&a,1m,7a)	$4 \cdot 10^{-10}$
2xli_B-16_B-17	1a	2=(1c)	1.0	1=(1a,1L,&a,1m,7a)	$2 \cdot 10^{-19}$
7st2_3-1198_3-1199	1a	2=(1c)	1.0	1=(1a,1L,&a,1m,7a)	$1 \cdot 10^{-10}$
3u56_B-2182_B-2183	1a	2=(1c)	1.0	1=(1a,1L,&a,1m,7a)	$10 \cdot 10^{-11}$
2eet_A-20_A-21	1a	2=(1c)	1.0	1=(1a,1L,&a,1m,7a)	$2 \cdot 10^{-11}$
7st2_3-719_3-720	1a	2=(1c)	1.0	1=(1a,1L,&a,1m,7a)	$2 \cdot 10^{-11}$
7st2_3-1518_3-1519	1L	2=(1c)	1.0	1=(1a,1L,&a,1m,7a)	$8 \cdot 10^{-15}$
2qbz_X-154_X-155	3d	3=(1g)	1.0	-	-
3ger_A-53_A-54	5n	4=(9a,3a,7a)	1.0	-	-
3ger_A-15_A-16	1a	2=(1c)	1.0	1=(1a,1L,&a,1m,7a)	$2 \cdot 10^{-15}$

Table F: Suites for which the sugar pucker-pair determined by the Pperp criterion do not match the sugar pucker-pair of the sequence related conformers (red in the plots) or the RNAprecis predicted cluster does not match the sequence related conformer (orange in the plots) sorted by posterior probability.

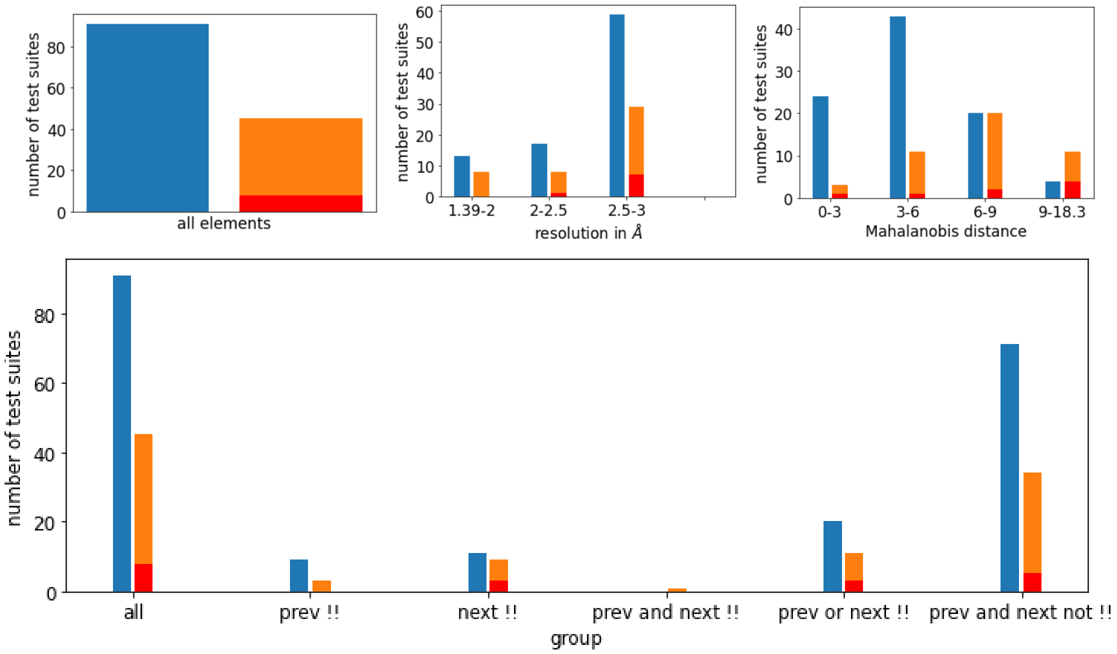

Fig Q: Relative distribution of agreements and disagreements of MINT-AGE cluster prediction with sequence related probable conformer classes for the test set LDTP33 with color scheme (reflecting blue for match, orange for mismatch and red for pucker-pair mismatch) from Section 3.4 in the main text). Top left: overall histogram. Top middle: histogram reflecting varying resolutions of PDB suites. Top right: histogram reflecting varying Mahanalobis distances from the predicted cluster. One can see that with increasing distance the rate of mismatches increases. This indicates that despite its limitations discussed in Remark 2.3 in the main text the distance is useful as an empirical indicator of prediction confidence. Bottom: histogram also reflecting dependence on whether previous, next or both suites have been !! or not. While false predictions are more likely if at least one neighboring suite is a !! suite, roughly half of the total false predictions are from suites without a neighboring !! suite.

E.2 Results for the test data set LDTP32

LDTP32: matches

name	p. answer	RNAprecis	p_{post}
7f5s_LI-1639_LI-1640	1b	**5**=(1[,1b,!!)	0.7
7f5s_LI-2510_LI-2511	3b	**3**=(3b)	0.84
7st2_3-1501_3-1502	7r	**4**=(7r)	0.89
7xny_Sc-47_Sc-48	1[	**1**=(1b,1[)	0.93
4lvy_A-54_A-55	1b	**1**=(1b,1[)	0.94
7f5s_LI-4487_LI-4488	1[	**1**=(1b,1[)	0.95
7st2_2-23_2-24	1b	**5**=(1[,1b,!!)	0.95
2qbz_X-45_X-46	1[	**5**=(1[,1b,!!)	0.96
7st2_2-39_2-40	1b	**1**=(1b,1[)	0.99
5fk1_A-67_A-68	1b	**1**=(1b,1[)	0.99
7f5s_LI-4337_LI-4338	1b	**1**=(1b,1[)	1.0
3u4m_B-2115_B-2116	1o	**8**=(1o)	1.0
3gx5_A-18_A-19	1b	**1**=(1b,1[)	1.0
4z4g_B-15_B-16	1b	**1**=(1b,1[)	1.0
7st2_3-883_3-884	1b	**1**=(1b,1[)	1.0
7st2_3-1431_3-1432	1b	**1**=(1b,1[)	1.0
4qg3_B-2125_B-2126	3b	**3**=(3b)	1.0
2eev_A-61_A-62	1t	**7**=(1t)	1.0
7f5s_LI-2738_LI-2739	7p	**9**=(7p)	1.0
7st2_2-43_2-44	7p	**14**=(7p)	1.0
7st2_3-6_3-7	1b	**5**=(1[,1b,!!)	1.0
7st2_3-558_3-559	5z	**2**=(5z)	1.0
7st2_3-450_3-451	5p	**12**=(5p)	1.0
7st2_3-720_3-721	5z	**2**=(5z)	1.0
7f5s_LI-4559_LI-4560	1z	**6**=(1z)	1.0
7st2_3-888_3-889	5z	**2**=(5z)	1.0
3umy_B-2144_B-2145	1z	**6**=(1z)	1.0
2qbz_X-100_X-101	5p	**11**=(5p)	1.0
2qbz_X-85_X-86	5p	**12**=(5p)	1.0
8eiu_A-1156_A-1157	1z	**6**=(1z)	1.0

LDTP32: pucker mismatches

name	p. answer	p_1	p_2	RNAprecis	p_{post}
7xny_SX-2463_SX-2464	1a	4.48	2.35	1=(1b,1[)	0.99
7st2_3-818_3-819	2u	3.74	1.11	11=(5p)	1.0
7st2_3-561_3-562	0b	3.65	0.55	3=(3b)	1.0
1c0a_B-636_B-637	1a	4.45	1.1	12=(5p)	1.0
7st2_3-280_3-281	2[	4.29	1.81	3=(3b)	1.0

LDTP32 conformer mismatches

name	p. answer	RNAprecis	p_{post}	first match	p_{post}
7st2_2-11_2-12	1b	7=(1t)	0.62	1=(1b,1[)	0.38
8eiu_A-12_A-13	1[	4=(7r)	1.0	1=(1b,1[)	$4 \cdot 10^{-6}$
7st2_3-732_3-733	1b	4=(7r)	1.0	1=(1b,1[)	$8 \cdot 10^{-10}$

Table G: Suites for which the RNAprecis predicted cluster matches the sequence related conformer (blue in the plots), the sugar pucker-pair determined by the Pperp criterion do not match the sugar pucker-pair of the sequence related conformers (red in the plots), or the RNAprecis predicted cluster does not match the sequence related conformer (orange in the plots); each sorted by posterior probability.

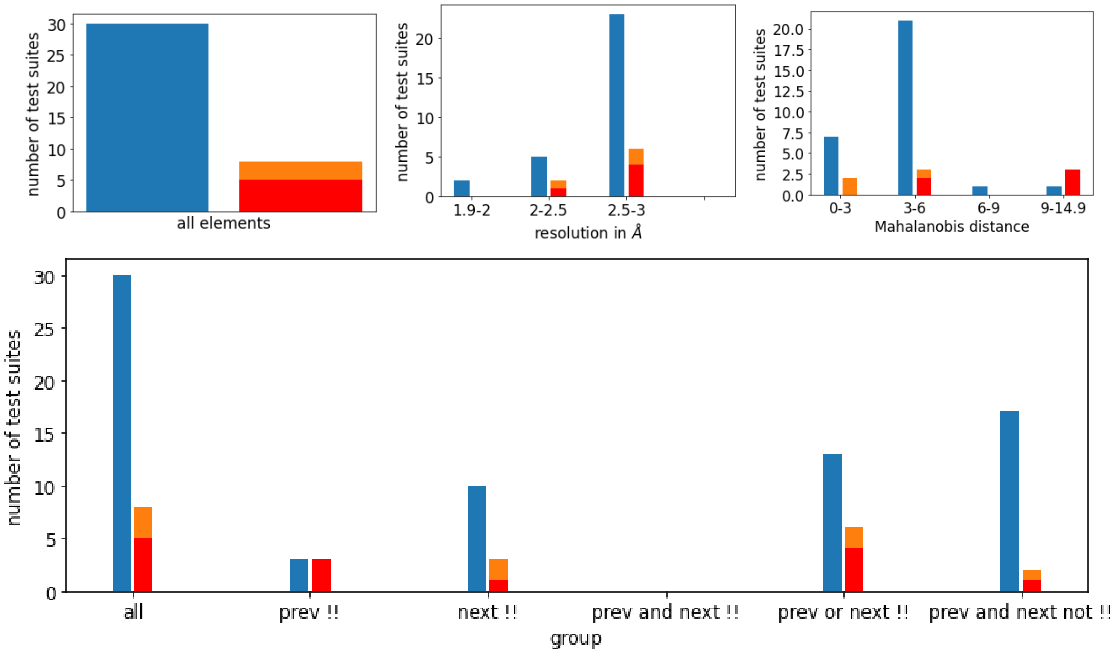

Fig R: Relative distribution of agreements and disagreements of MINT-AGE cluster prediction with sequence related probable conformer classes for the test set LDT32 with color scheme (reflecting blue for match, orange for mismatch and red for pucker-pair mismatch) from Section 3.4 in the main text). Top left: overall histogram. Top middle: histogram reflecting varying resolutions of PDB suites. Top right: histogram reflecting varying Mahanalobis distances from the predicted cluster. One can see that with increasing distance the rate of mismatches increases. This indicates that despite its limitations discussed in Remark 2.3 in the main text the distance is useful as an empirical indicator of prediction confidence. Bottom: histogram also reflecting dependence on whether previous, next or both suites have been !! or not.

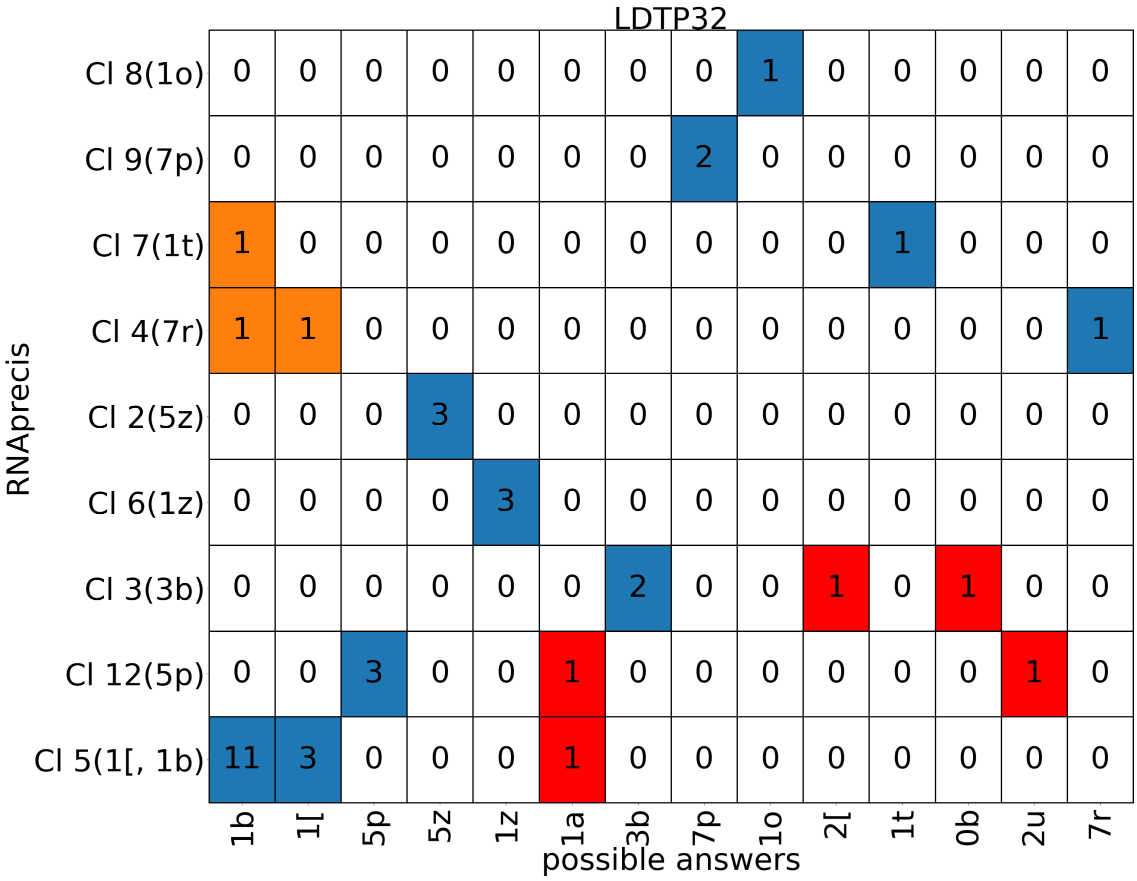

Fig S: Classification matrix listing counts of probable sequence related conformation classes (horizontal axis) versus MINT-AGE clusters (vertical, with conformer classes in parentheses from MINT-AGE training, see Fig J) for the low detail training data set of sugar pucker-pair LDP32 with colors (from Section 3.4 in the main text): blue for match, orange for mismatch and red for pucker-pair mismatch. Columns listing multiple suite conformers are from sites solved in different conformations across sequence-related models, and any of the sequence-related conformations were accepted as a matching predictions. As pointed out in Section 3.4, the **1a**,**3g**,**8d** and **3d**,**7p** suites have multiple sequence-related conformations. This means that the counts in these two columns are inflated.

E.3 Results for the test data set LDTP23

LDTP23: matches

name	p. answer	RNAprecis	p_{post}
7f5s_LI-4556_LI-4557	0a	**6**=(0a)	0.52
7f5s_LI-2741_LI-2742	6g	**4**=(6g,4g)	0.92
3gx5_A-35_A-36	0a	**2**=(0a,#a,4a)	0.93
7st2_3-535_3-536	2a	**1**=(2a)	0.94
5fk1_A-46_A-47	4g	**4**=(6g,4g)	0.96
2qbz_X-34_X-35	6g	**4**=(6g,4g)	0.97
7st2_3-1498_3-1499	6g	**4**=(6g,4g)	0.97
7f5s_LI-4465_LI-4466	4g	**15**=(4g)	0.98
7st2_3-1394_3-1395	2a	**1**=(2a)	0.99
7f5s_LI-4331_LI-4332	6d	**13**=(6d)	0.99
4kze_R-61_R-62	8d	**5**=(8d,4d)	0.99
6y0t_A-2647_A-2648	0a	**6**=(0a)	1.0
7f5s_LI-2834_LI-2835	6j	**10**=(6j)	1.0
7st2_3-960_3-961	6d	**8**=(6d)	1.0
7st2_3-733_3-734	4d	**5**=(8d,4d)	1.0
8eiu_A-508_A-509	6g	**4**=(6g,4g)	1.0
7st2_3-1506_3-1507	2h	**14**=(2h)	1.0
6qis_G-5_G-6	2a	**1**=(2a)	1.0
4qg3_B-2172_B-2173	2a	**1**=(2a)	1.0
5npm_B-7_B-8	8d	**5**=(8d,4d)	1.0
7f5s_LI-4354_LI-4355	6d	**8**=(6d)	1.0
7st2_2-12_2-13	4a	**7**=(4a)	1.0
7f5s_LI-349_LI-350	2a	**1**=(2a)	1.0
6b3k_R-28_R-29	2a	**1**=(2a)	1.0
7f5s_LI-4221_LI-4222	6j	**10**=(6j)	1.0
7st2_3-687_3-688	0i	**9**=(0i)	1.0
5fk1_A-20_A-21	0i	**9**=(0i)	1.0
7f5s_LI-3747_LI-3748	6j	**10**=(6j)	1.0

LDTP23: pucker mismatches

name	p. answer	p_1	p_2	RNAprecis	p_{post}
4xw7_A-27_A-28	1a	2.17	4.33	12=(4n)	0.73
7f5s_LI-2464_LI-2465	1a	2.46	4.34	12=(4n)	0.93
7st2_3-817_3-818	6p	0.55	3.74	5=(8d,4d)	0.99
1c0a_B-634_B-635	1a	0.47	4.61	2=(0a,#a,4a)	1.0
2qbz_X-138_X-139	&a	0.95	4.49	12=(4n)	1.0
7st2_3-560_3-561	6p	1.72	3.65	8=(6d)	1.0
2qbz_X-47_X-48	6p	1.87	4.38	2=(0a,#a,4a)	1.0

LDTP23 conformer mismatches

name	p. answer	RNAprecis	p_{post}	first match	p_{post}
7st2_3-115_3-116	6g	16=(4g,4a)	0.79	4=(6g,4g)	0.21
7f5s_LI-151_LI-152	0a	4=(6g,4g)	0.81	2=(0a,#a,4a)	0.04
7st2_3-305_3-306	6g	10=(6j)	0.98	4=(6g,4g)	0.02
8eiu_A-345_A-346	6n	4=(6g,4g)	0.99	3=(6n,0i,6j)	0.01
6b3k_R-57_R-58	4n	4=(6g,4g)	0.99	12=(4n)	$8 \cdot 10^{-3}$
6xh0_D-35_D-36	8d	1=(2a)	1.0	5=(8d,4d)	$2 \cdot 10^{-3}$
7st2_3-641_3-642	0a	16=(4g,4a)	1.0	2=(0a,#a,4a)	$2 \cdot 10^{-6}$
2qbz_X-29_X-30	4g	2=(0a,#a,4a)	1.0	4=(6g,4g)	$8 \cdot 10^{-5}$
2qbz_X-101_X-102	2g	1=(2a)	1.0	11=(2g,8d)	$8 \cdot 10^{-46}$
2qbz_X-87_X-88	8d	13=(6d)	1.0	5=(8d,4d)	$3 \cdot 10^{-8}$
7st2_3-518_3-519	6n	4=(6g,4g)	1.0	3=(6n,0i,6j)	$4 \cdot 10^{-7}$
7st2_3-753_3-754	6n	4=(6g,4g)	1.0	3=(6n,0i,6j)	$3 \cdot 10^{-21}$
4z4g_B-16_B-17	2a	14=(2h)	1.0	1=(2a)	$6 \cdot 10^{-16}$
3ger_A-63_A-64	6n	2=(0a,#a,4a)	1.0	3=(6n,0i,6j)	$10 \cdot 10^{-119}$
3gx5_A-9_A-10	2a	2=(0a,#a,4a)	1.0	1=(2a)	$7 \cdot 10^{-21}$

Table H: Suites for which the RNAprecis predicted cluster matches the sequence related conformer (blue in the plots), the sugar pucker-pair determined by the Pperp criterion do not match the sugar pucker-pair of the sequence related conformers (red in the plots), or the RNAprecis predicted cluster does not match the sequence related conformer (orange in the plots); each sorted by posterior probability.

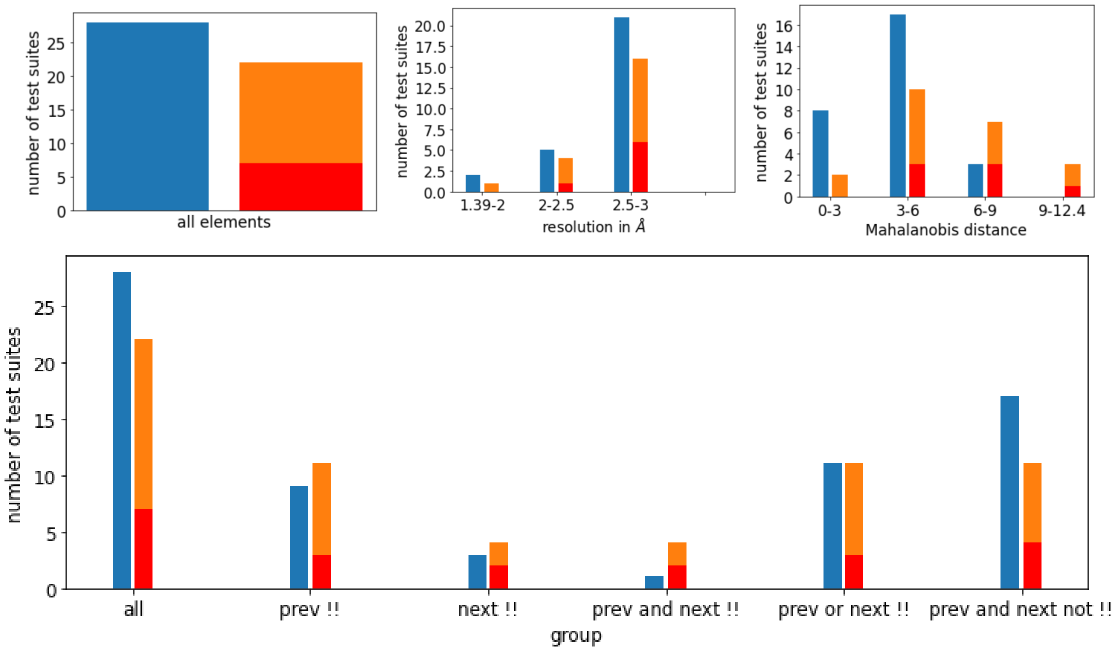

Fig T: Relative distribution of agreements and disagreements of MINT-AGE cluster prediction with sequence related probable conformer classes for the test set LDT23 with color scheme (reflecting blue for match, orange for mismatch and red for pucker-pair mismatch) from Section 3.4 in the main text). Top left: overall histogram. Top middle: histogram reflecting varying resolutions of PDB suites. Top right: histogram reflecting varying Mahanalobis distances from the predicted cluster. One can see that with increasing distance the rate of mismatches increases. This indicates that despite its limitations discussed in Remark 2.3 in the main text the distance is useful as an empirical indicator of prediction confidence. Bottom: histogram also reflecting dependence on whether previous, next or both suites have been !! or not.

LDTP23

RNAprecis	2a	6g	0a	8d	6n	6d	6p	4g	1a	6j	0i	2g	4n	4d	4a	&a	2h
Cl 7(4a)	0	0	0	0	0	0	0	0	0	0	0	0	0	0	1	0	0
Cl 15(4g)	0	0	0	0	0	0	0	1	0	0	0	0	0	0	0	0	0
Cl 16(4g, 4a)	0	1	1	0	0	0	0	0	0	0	0	0	0	0	0	0	0
Cl 6(0a)	0	0	2	0	0	0	0	0	0	0	0	0	0	0	0	0	0
Cl 9(0i)	0	0	0	0	0	0	0	0	0	0	2	0	0	0	0	0	0
Cl 14(2h)	1	0	0	0	0	0	0	0	0	0	0	0	0	0	0	0	1
Cl 12(4n)	0	0	0	0	0	0	0	0	2	0	0	0	0	0	0	1	0
Cl 10(6j)	0	1	0	0	0	0	0	0	0	3	0	0	0	0	0	0	0
Cl 5(8d, 4d)	0	0	0	2	0	0	1	0	0	0	0	0	0	1	0	0	0
Cl 13(6d)	0	0	0	1	0	3	1	0	0	0	0	0	0	0	0	0	0
Cl 2(0a, #a, 4a)	1	0	1	0	1	0	1	1	1	0	0	0	0	0	0	0	0
Cl 1(2a)	6	0	0	1	0	0	0	0	0	0	0	1	0	0	0	0	0
Cl 4(6g, 4g)	0	4	1	0	3	0	0	1	0	0	0	0	1	0	0	0	0

possible answers

Fig U: Classification matrix listing counts of probable sequence related conformation classes (horizontal axis) versus MINT-AGE clusters (vertical, with conformer classes in parentheses from MINT-AGE training, see Fig K) for the low detail training data set of sugar pucker-pair LDP23 with colors (from Section 3.4 in the main text): blue for match, orange for mismatch and red for pucker-pair mismatch. Columns listing multiple suite conformers are from sites solved in different conformations across sequence-related models, and any of the sequence-related conformations were accepted as a matching predictions. Columns listing multiple suite conformers are from sites solved in different conformations across sequence-related models, and any of the sequence-related conformations were accepted as a matching predictions.

E.4 Results for the test data set LDTP22

LDTP22: matches

name	p. answer	RNAprecis	p_{post}
7f5s_LI-1640_LI-1641	4p	**3**=(4p)	0.88
5npm_B-14_B-15	4p	**3**=(4p)	0.97
6xh1_D-33_D-34	2[	**6**=(2u,2o,2[)	0.98
3ger_A-62_A-63	0b	**8**=(0b)	0.99
7f5s_LI-4379_LI-4380	4b	**10**=(4b)	1.0
8eiu_A-344_A-345	2[	**1**=(2[)	1.0
7st2_3-752_3-753	4b	**4**=(4b)	1.0
7f5s_LL-37_LL-38	6p	**2**=(6p,!!)	1.0
7st2_2-14_2-15	2[	**1**=(2[)	1.0
7f5s_LI-1641_LI-1642	0b	**9**=(0b)	1.0
7st2_3-1181_3-1182	4b	**7**=(4b,0b)	1.0
3ger_A-48_A-49	2[	**1**=(2[)	1.0
7xny_Sc-22_Sc-23	4p	**3**=(4p)	1.0
2qbz_X-86_X-87	2[	**1**=(2[)	1.0
7f5s_LI-1881_LI-1882	2z	**5**=(2z)	1.0
7st2_3-559_3-560	6p	**2**=(6p,!!)	1.0

LDTP22: pucker mismatches

name	p. answer	p_1	p_2	RNAprecis	p_{post}
7st2_3-1398_3-1399	3b	2.48	0.65	9=(0b)	0.68
7st2_3-982_3-983	6n	0.27	1.98	1=(2[)	1.0

LDTP22 conformer mismatches

name	p. answer	RNAprecis	p_{post}	first match	p_{post}
3gx5_A-19_A-20	4b	1=(2[)	0.53	4=(4b)	0.47
3gx5_A-63_A-64	2z	6=(2u,2o,2[)	0.54	5=(2z)	0.46

Table I: Suites for which the RNAprecis predicted cluster matches the sequence related conformer (blue in the plots), the sugar pucker-pair determined by the Pperp criterion do not match the sugar pucker-pair of the sequence related conformers (red in the plots), or the RNAprecis predicted cluster does not match the sequence related conformer (orange in the plots); each sorted by posterior probability.

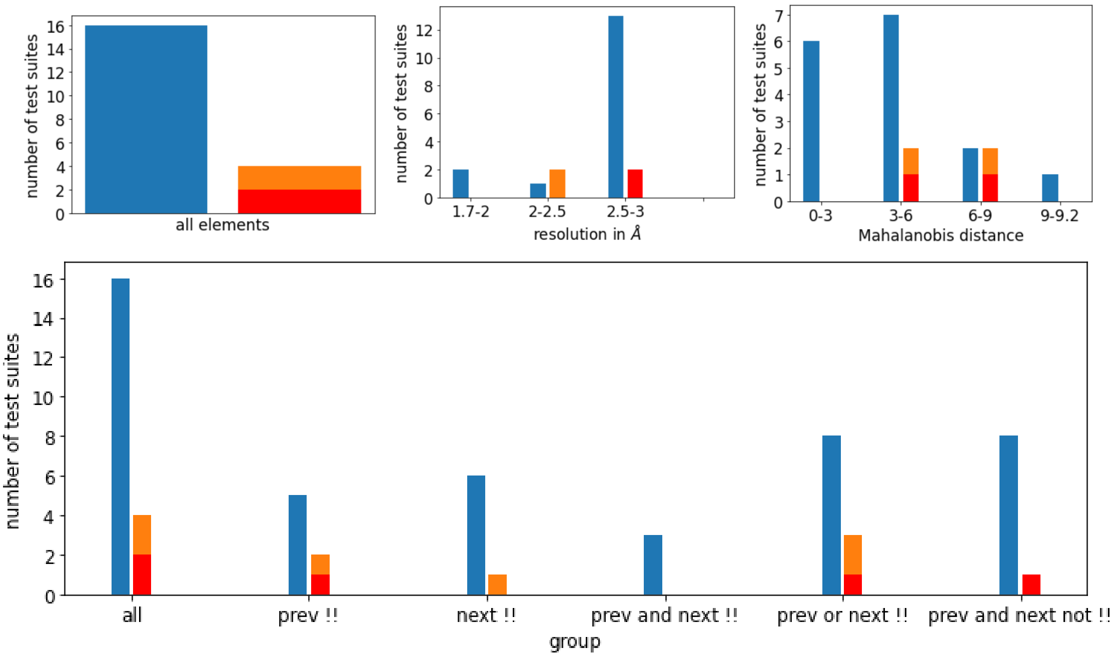

Fig V: Relative distribution of agreements and disagreements of MINT-AGE cluster prediction with sequence related probable conformer classes for the test set LDT22 with color scheme (reflecting blue for match, orange for mismatch and red for pucker-pair mismatch) from Section 3.4 in the main text). Top left: overall histogram. Top middle: histogram reflecting varying resolutions of PDB suites. Top right: histogram reflecting varying Mahanalobis distances from the predicted cluster. One can see that with increasing distance the rate of mismatches increases. This indicates that despite its limitations discussed in Remark 2.3 in the main text the distance is useful as an empirical indicator of prediction confidence. Bottom: histogram also reflecting dependence on whether previous, next or both suites have been !! or not.

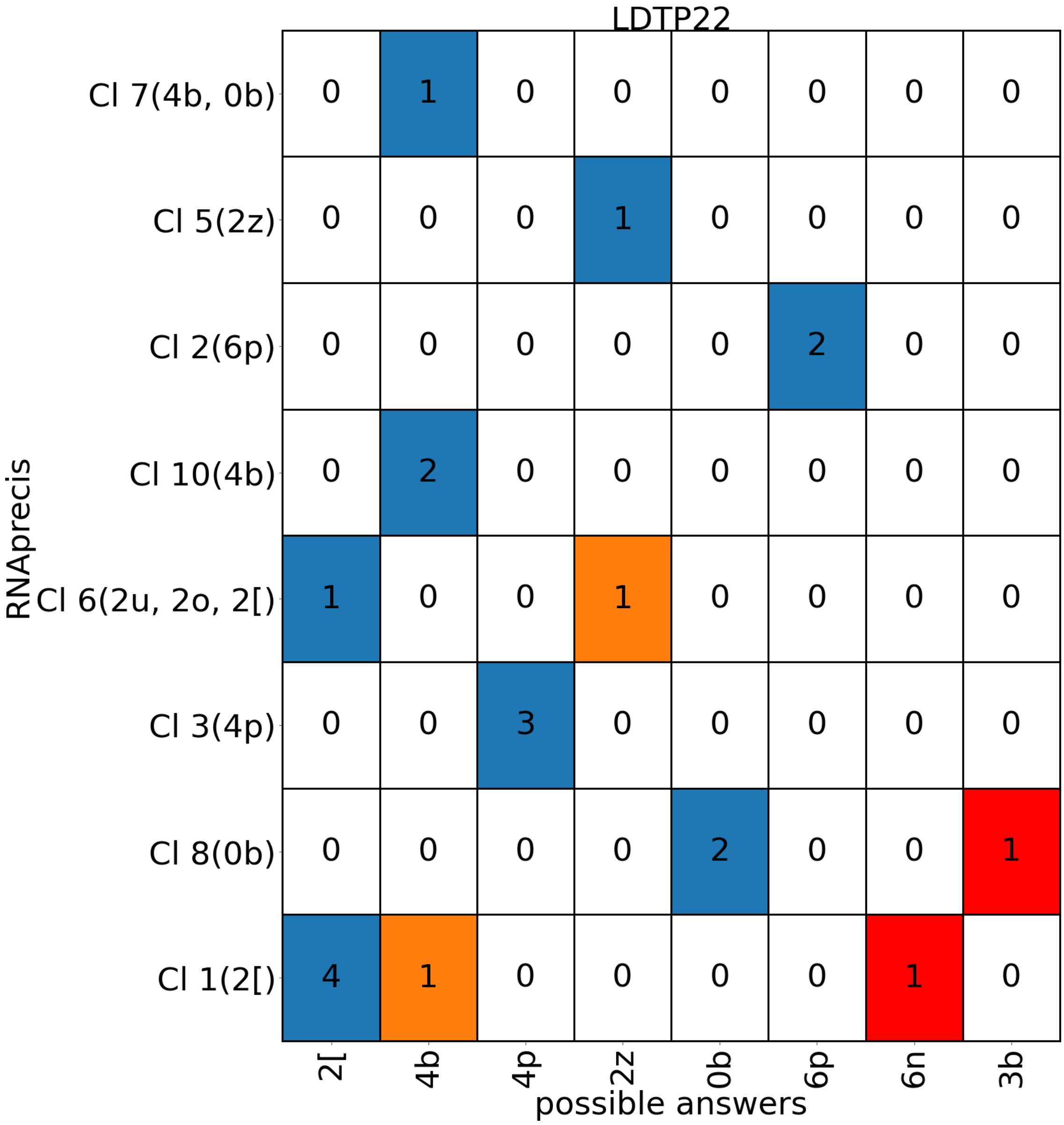

	2[	4b	4p	2z	0b	6p	6n	3b
Cl 7(4b, 0b)	0	1	0	0	0	0	0	0
Cl 5(2z)	0	0	0	1	0	0	0	0
Cl 2(6p)	0	0	0	0	0	2	0	0
Cl 10(4b)	0	2	0	0	0	0	0	0
Cl 6(2u, 2o, 2[)	1	0	0	1	0	0	0	0
Cl 3(4p)	0	0	3	0	0	0	0	0
Cl 8(0b)	0	0	0	0	2	0	0	1
Cl 1(2[)	4	1	0	0	0	0	1	0

Fig W: Classification matrix listing counts of probable sequence related conformation classes (horizontal axis) versus MINT-AGE clusters (vertical, with conformer classes in parentheses from MINT-AGE training, see Fig L) for the low detail training data set of sugar pucker-pair LDP22 with colors (from Section 3.4 in the main text): blue for match, orange for mismatch and red for pucker-pair mismatch. Columns listing multiple suite conformers are from sites solved in different conformations across sequence-related models, and any of the sequence-related conformations were accepted as a matching predictions. Columns listing multiple suite conformers are from sites solved in different conformations across sequence-related models, and any of the sequence-related conformations were accepted as a matching predictions.

E.5 Results for the test data set 8b0xP33

8b0xP33: matches

name	p. answer	RNAprecis	p_{post}
7st2_1-2330_1-2331	1c	**2**=(1c)	0.52
7st2_1-382_1-383	1c	**2**=(1c)	0.53
7st2_1-2817_1-2818	1c	**2**=(1c)	0.91
7st2_1-2553_1-2554	1e	**7**=(1e,1f)	0.94
7st2_1-402_1-403	1c	**2**=(1c)	0.99
7st2_1-1775_1-1776	1c	**2**=(1c)	0.99
7st2_1-229_1-230	1c	**2**=(1c)	1.0
7st2_1-1638_1-1639	1c	**2**=(1c)	1.0
7st2_1-1503_1-1504	1c	**2**=(1c)	1.0
7st2_1-1436_1-1437	1c	**2**=(1c)	1.0
7st2_1-567_1-568	1c	**2**=(1c)	1.0
7st2_1-254_1-255	1e	**5**=(1e)	1.0
7st2_1-2278_1-2279	1c	**2**=(1c)	1.0
7st2_1-2444_1-2445	1c	**2**=(1c)	1.0
7st2_1-1375_1-1376	1c	**2**=(1c)	1.0
7st2_1-1002_1-1003	1c	**2**=(1c)	1.0
8eiu_a-1790_a-1791	7a	**4**=(9a,3a,7a)	1.0
7st2_1-2715_1-2716	1c	**2**=(1c)	1.0
7st2_1-561_1-562	7d	**8**=(7d)	1.0
8eiu_a-2317_a-2318	1a	**1**=(1a,1L,&a,1m,7a)	1.0
7st2_1-998_1-999	1c	**2**=(1c)	1.0
7st2_1-694_1-695	1c	**2**=(1c)	1.0
7st2_1-906_1-907	1c	**2**=(1c)	1.0
7st2_1-2242_1-2243	1c	**2**=(1c)	1.0
7st2_1-818_1-819	1e	**5**=(1e)	1.0
7st2_1-2349_1-2350	1c	**2**=(1c)	1.0
7st2_1-2763_1-2764	7a	**4**=(9a,3a,7a)	1.0
7st2_1-1138_1-1139	1c	**2**=(1c)	1.0
7st2_1-868_1-869	1c	**2**=(1c)	1.0
7st2_1-109_1-110	1c	**2**=(1c)	1.0
7st2_1-940_1-941	1e	**7**=(1e,1f)	1.0
7st2_1-2479_1-2480	1c	**2**=(1c)	1.0
7st2_1-1902_1-1903	1c	**2**=(1c)	1.0
7st2_1-293_1-294	1f	**11**=(1f)	1.0
7st2_1-219_1-220	1a	**1**=(1a,1L,&a,1m,7a)	1.0
7st2_1-2048_1-2049	1c	**2**=(1c)	1.0
8eiu_a-2433_a-2434	7a	**4**=(9a,3a,7a)	1.0
8eiu_a-2079_a-2080	1c	**2**=(1c)	1.0
7st2_1-621_1-622	1c	**2**=(1c)	1.0
7st2_1-251_1-252	1c	**2**=(1c)	1.0
7st2_1-500_1-501	1g	**3**=(1g)	1.0
7st2_1-2360_1-2361	1c	**2**=(1c)	1.0
7st2_1-476_1-477	1g	**3**=(1g)	1.0
7st2_1-2475_1-2476	7d	**8**=(7d)	1.0
7st2_1-1671_1-1672	1g	**3**=(1g)	1.0
7st2_1-423_1-424	5j	**10**=(5j)	1.0
7st2_1-2563_1-2564	1g	**3**=(1g)	1.0
7st2_1-1254_1-1255	5d	**9**=(5d)	1.0
7st2_1-1926_1-1927	1g	**3**=(1g)	1.0
7st2_1-2871_1-2872	3a	**4**=(9a,3a,7a)	1.0
7st2_1-2890_1-2891	1f	**11**=(1f)	1.0
7st2_1-148_1-149	7d	**8**=(7d)	1.0
7st2_1-1367_1-1368	1c	**2**=(1c)	1.0

Table J: Suites for which the RNAprecis predicted cluster matches the sequence related conformer (blue in the plots) sorted by posterior probability.

8b0xP33: pucker mismatches

name	p. answer	p_1	p_2	RNAprecis	p_{post}
7st2_1-1418_1-1419	1b	4.78	3.78	5=(1e)	1.0
7st2_1-2807_1-2808	1b	4.57	3.36	2=(1c)	1.0
7st2_1-395_1-396	4g	3.89	4.7	1=(1a,1L,&a,1m,7a)	1.0
7st2_1-394_1-395	1b	4.74	3.89	2=(1c)	1.0
7st2_1-228_1-229	6n	4.05	4.57	4=(9a,3a,7a)	1.0
7st2_1-857_1-858	1b	4.63	2.96	2=(1c)	1.0
7st2_1-1457_1-1458	3b	4.64	3.17	4=(9a,3a,7a)	1.0
7st2_1-1559_1-1560	2a	3.93	4.61	4=(9a,3a,7a)	1.0
7st2_1-1626_1-1627	0a	3.86	4.67	4=(9a,3a,7a)	1.0
7st2_1-1856_1-1857	1b	4.64	4.12	2=(1c)	1.0
7st2_1-1883_1-1884	1b	4.44	3.99	2=(1c)	1.0
7st2_1-1900_1-1901	6j	3.63	4.5	10=(5j)	1.0
7st2_1-2808_1-2809	0a	3.36	4.61	4=(9a,3a,7a)	1.0

8b0xP33: conformer mismatches

name	p. answer	RNAprecis	p_{post}	first match	p_{post}
7st2_1-95_1-96	1c	7=(1e,1f)	0.64	2=(1c)	0.36
8eiu_a-1749_a-1750	1a	2=(1c)	0.68	1=(1a,1L,&a,1m,7a)	0.32
7st2_1-333_1-334	1a	2=(1c)	0.77	1=(1a,1L,&a,1m,7a)	0.23
7st2_1-57_1-58	1c	1=(1a,1L,&a,1m,7a)	0.79	2=(1c)	0.21
8eiu_a-1240_a-1241	&a	5=(1e)	0.92	1=(1a,1L,&a,1m,7a)	$2 \cdot 10^{-28}$
8eiu_a-2534_a-2535	1f	2=(1c)	0.96	7=(1e,1f)	0.04
7st2_1-1477_1-1478	1a	2=(1c)	0.98	1=(1a,1L,&a,1m,7a)	0.02
8eiu_a-44_a-45	1f	2=(1c)	0.98	7=(1e,1f)	0.02
7st2_1-29_1-30	1a	2=(1c)	0.98	1=(1a,1L,&a,1m,7a)	0.02
7st2_1-2326_1-2327	1a	6=(1f)	0.99	1=(1a,1L,&a,1m,7a)	$4 \cdot 10^{-9}$
7st2_1-2473_1-2474	1a	2=(1c)	1.0	1=(1a,1L,&a,1m,7a)	$2 \cdot 10^{-3}$
7st2_1-516_1-517	1a	2=(1c)	1.0	1=(1a,1L,&a,1m,7a)	$1 \cdot 10^{-3}$
7st2_1-2886_1-2887	1a	2=(1c)	1.0	1=(1a,1L,&a,1m,7a)	$3 \cdot 10^{-4}$
8eiu_a-1873_a-1874	1a	2=(1c)	1.0	1=(1a,1L,&a,1m,7a)	$1 \cdot 10^{-4}$
8eiu_a-537_a-538	7a	5=(1e)	1.0	4=(9a,3a,7a)	$10 \cdot 10^{-6}$
7st2_1-1154_1-1155	1L	4=(9a,3a,7a)	1.0	1=(1a,1L,&a,1m,7a)	$2 \cdot 10^{-8}$
8eiu_a-130_a-131	1e	2=(1c)	1.0	7=(1e,1f)	$2 \cdot 10^{-8}$
7st2_1-2564_1-2565	1a	2=(1c)	1.0	1=(1a,1L,&a,1m,7a)	$4 \cdot 10^{-9}$
7st2_1-499_1-500	1a	2=(1c)	1.0	1=(1a,1L,&a,1m,7a)	$2 \cdot 10^{-9}$
8eiu_a-2474_a-2475	1a	2=(1c)	1.0	1=(1a,1L,&a,1m,7a)	$1 \cdot 10^{-9}$
7st2_1-2822_1-2823	1g	8=(7d)	1.0	3=(1g)	$6 \cdot 10^{-10}$
7st2_1-633_1-634	1a	2=(1c)	1.0	1=(1a,1L,&a,1m,7a)	$7 \cdot 10^{-12}$
7st2_1-2858_1-2859	1a	2=(1c)	1.0	1=(1a,1L,&a,1m,7a)	$10 \cdot 10^{-13}$
7st2_1-1185_1-1186	3d	3=(1g)	1.0	-	-
7st2_1-2202_1-2203	3d	3=(1g)	1.0	-	-
7st2_1-464_1-465	1a	2=(1c)	1.0	1=(1a,1L,&a,1m,7a)	$8 \cdot 10^{-14}$
7st2_1-45_1-46	5n	4=(9a,3a,7a)	1.0	-	-
7st2_1-1004_1-1005	3g	3=(1g)	1.0	-	-
7st2_1-1756_1-1757	3d	8=(7d)	1.0	-	-
7st2_1-1358_1-1359	&a	4=(9a,3a,7a)	1.0	1=(1a,1L,&a,1m,7a)	$4 \cdot 10^{-21}$
7st2_1-218_1-219	1a	2=(1c)	1.0	1=(1a,1L,&a,1m,7a)	$4 \cdot 10^{-18}$
7st2_1-466_1-467	1a	2=(1c)	1.0	1=(1a,1L,&a,1m,7a)	$1 \cdot 10^{-19}$

Table K: Suites for which the sugar pucker-pair determined by the Pperp criterion do not match the sugar pucker-pair of the sequence related conformers (red in the plots) or the RNAprecis predicted cluster does not match the sequence related conformer (orange in the plots) sorted by posterior probability.

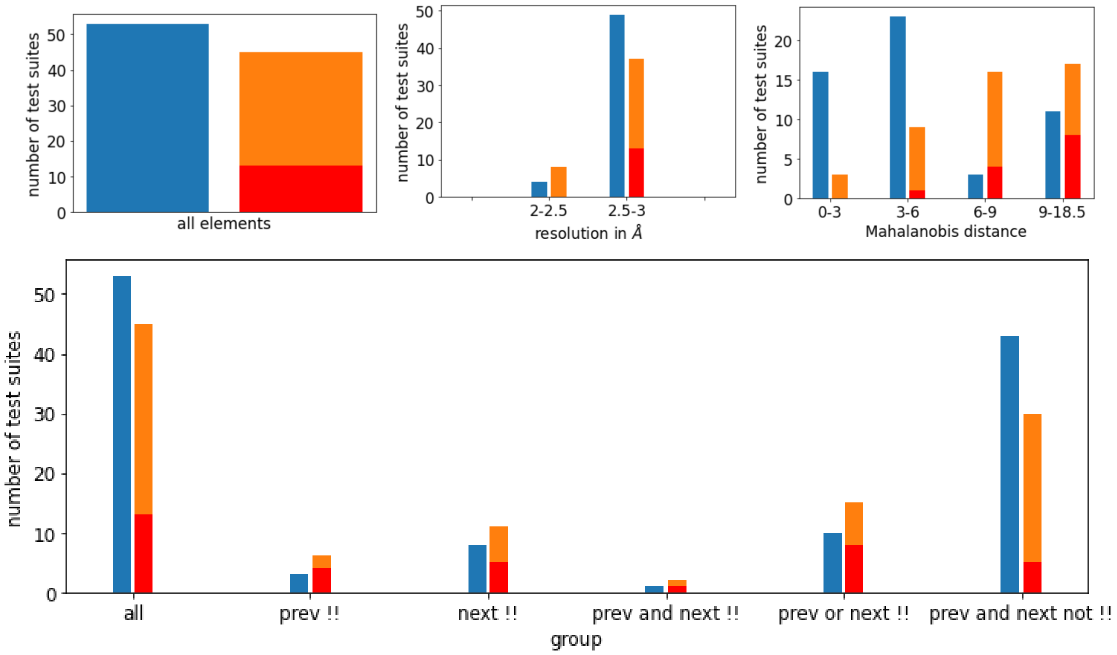

Fig X: Relative distribution of agreements and disagreements of MINT-AGE cluster prediction with sequence related probable conformer classes for the test set LDTP33 with color scheme (reflecting blue for match, orange for mismatch and red for pucker-pair mismatch) from Section 3.4 in the main text). Top left: overall histogram. Top middle: histogram reflecting varying resolutions of PDB suites. Top right: histogram reflecting varying Mahanalobis distances from the predicted cluster. One can see that with increasing distance the rate of mismatches increases. This indicates that despite its limitations discussed in Remark 2.3 in the main text the distance is useful as an empirical indicator of prediction confidence. Bottom: histogram also reflecting dependence on whether previous, next or both suites have been !! or not. While false predictions are more likely if at least one neighboring suite is a !! suite, roughly half of the total false predictions are from suites without a neighboring !! suite.

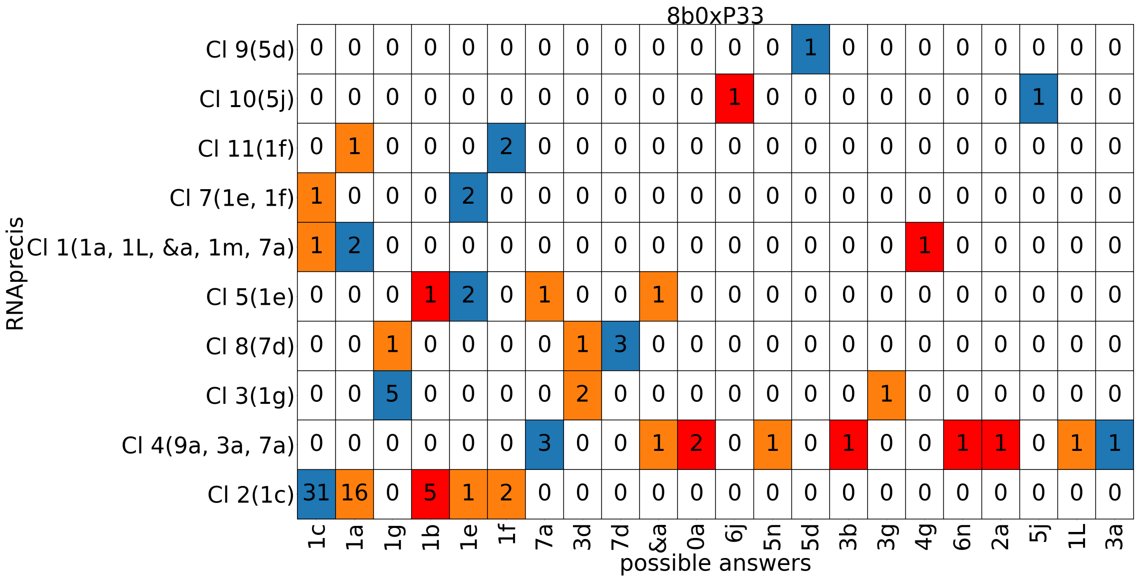

Fig Y: Classification matrix listing counts of probable sequence related conformation classes (horizontal axis) versus MINT-AGE clusters (vertical, with conformer classes in parentheses from MINT-AGE training, see Fig I) for the low detail training data set of sugar pucker-pair LDP33 with colors (from Section 3.4 in the main text): blue for match, orange for mismatch and red for pucker-pair mismatch. Columns listing multiple suite conformers are from sites solved in different conformations across sequence-related models, and any of the sequence-related conformations were accepted as a matching predictions. Columns listing multiple suite conformers are from sites solved in different conformations across sequence-related models, and any of the sequence-related conformations were accepted as a matching predictions.

E.6 Results for the test data set 8b0xP32

8b0xP32: matches

name	p. answer	RNAprecis	p_{post}
7st2_1-684_1-685	7r	**4**=(7r)	1.0
7st2_1-83_1-84	3b	**3**=(3b)	1.0
7st2_1-2731_1-2732	1b	**1**=(1b,1[)	1.0
8eiu_a-2343_a-2344	1b	**1**=(1b,1[)	1.0
7st2_1-1426_1-1427	5z	**2**=(5z)	1.0
8eiu_a-1625_a-1626	1b	**1**=(1b,1[)	1.0
7st2_1-1235_1-1236	1b	**1**=(1b,1[)	1.0
7st2_1-528_1-529	1t	**7**=(1t)	1.0
7st2_1-1935_1-1936	1[	**5**=(1[,1b,!!)	1.0
8eiu_a-864_a-865	1b	**1**=(1b,1[)	1.0
7st2_1-1246_1-1247	1t	**7**=(1t)	1.0
7st2_1-1785_1-1786	1z	**6**=(1z)	1.0
7st2_1-264_1-265	1z	**6**=(1z)	1.0
7st2_1-1129_1-1130	7p	**10**=(7p)	1.0
7st2_1-2571_1-2572	5p	**11**=(5p)	1.0
7st2_1-988_1-989	1t	**7**=(1t)	1.0
8eiu_a-2446_a-2447	7r	**4**=(7r)	1.0
7st2_1-1274_1-1275	1z	**6**=(1z)	1.0
7st2_1-1024_1-1025	5z	**2**=(5z)	1.0
7st2_1-1521_1-1522	5z	**2**=(5z)	1.0
7st2_1-1553_1-1554	1z	**6**=(1z)	1.0
7st2_1-1557_1-1558	5z	**2**=(5z)	1.0
7st2_1-1697_1-1698	5z	**2**=(5z)	1.0
7st2_1-972_1-973	5z	**2**=(5z)	1.0
7st2_1-799_1-800	5z	**2**=(5z)	1.0
7st2_1-1965_1-1966	5z	**2**=(5z)	1.0
7st2_1-1995_1-1996	5z	**2**=(5z)	1.0
7st2_1-240_1-241	5z	**2**=(5z)	1.0
7st2_1-203_1-204	5z	**2**=(5z)	1.0
7st2_1-198_1-199	1b	**5**=(1[,1b,!!)	1.0
7st2_1-99_1-100	3b	**3**=(3b)	1.0
7st2_1-1203_1-1204	5z	**2**=(5z)	1.0
7st2_1-1264_1-1265	5z	**2**=(5z)	1.0

8b0xP32: pucker mismatches

name	p. answer	p_1	p_2	RNAprecis	p_{post}
7st2_1-669_1-670	2[	3.93	0.57	1=(1b,1[)	1.0
7st2_1-1394_1-1395	1c	4.59	1.53	1=(1b,1[)	1.0
7st2_1-1211_1-1212	2[	4.34	1.97	3=(3b)	1.0

8b0xP32 conformer mismatches

name	p. answer	RNAprecis	p_{post}	first match	p_{post}
7st2_1-1633_1-1634	1z	9=(7p)	1.0	6=(1z)	$4 \cdot 10^{-5}$
7st2_1-1979_1-1980	1b	7=(1t)	1.0	1=(1b,1[)	$8 \cdot 10^{-6}$
7st2_1-1757_1-1758	5r	5=(1[,1b,!!)	1.0	-	-
7st2_1-1779_1-1780	5r	3=(3b)	1.0	-	-

Table L: Suites for which the RNAprecis predicted cluster matches the sequence related conformer (blue in the plots), the sugar pucker-pair determined by the Pperp criterion do not match the sugar pucker-pair of the sequence related conformers (red in the plots), or the RNAprecis predicted cluster does not match the sequence related conformer (orange in the plots); each sorted by posterior probability.

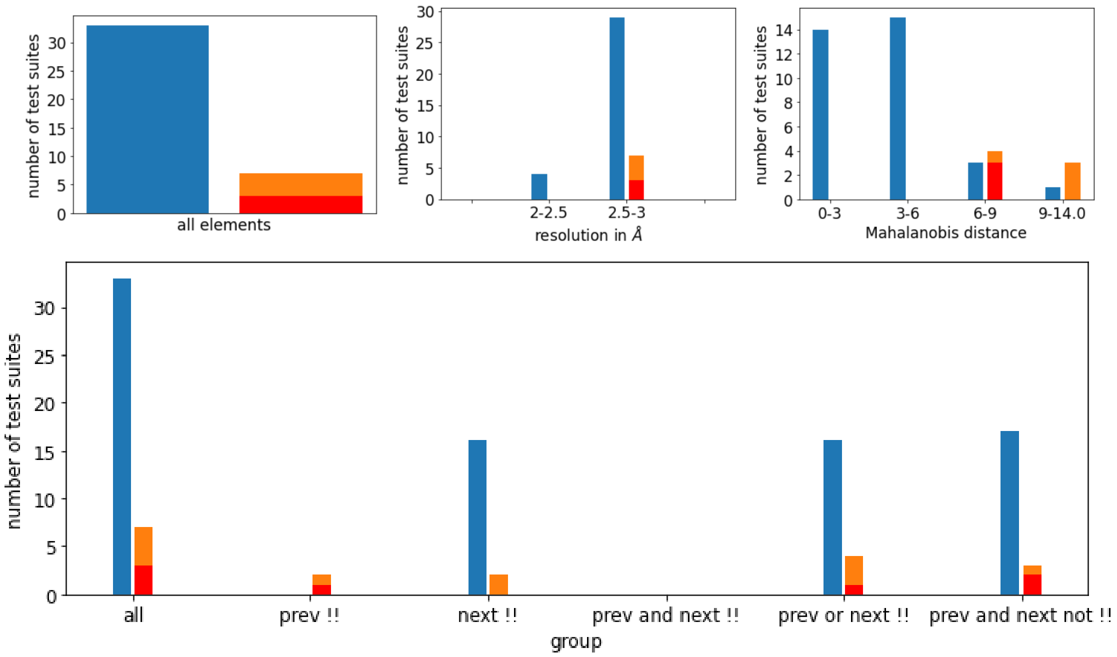

Fig Z: Relative distribution of agreements and disagreements of MINT-AGE cluster prediction with sequence related probable conformer classes for the test set LDT32 with color scheme (reflecting blue for match, orange for mismatch and red for pucker-pair mismatch) from Section 3.4 in the main text). Top left: overall histogram. Top middle: histogram reflecting varying resolutions of PDB suites. Top right: histogram reflecting varying Mahanalobis distances from the predicted cluster. One can see that with increasing distance the rate of mismatches increases. This indicates that despite its limitations discussed in Remark 2.3 in the main text the distance is useful as an empirical indicator of prediction confidence. Bottom: histogram also reflecting dependence on whether previous, next or both suites have been !! or not.

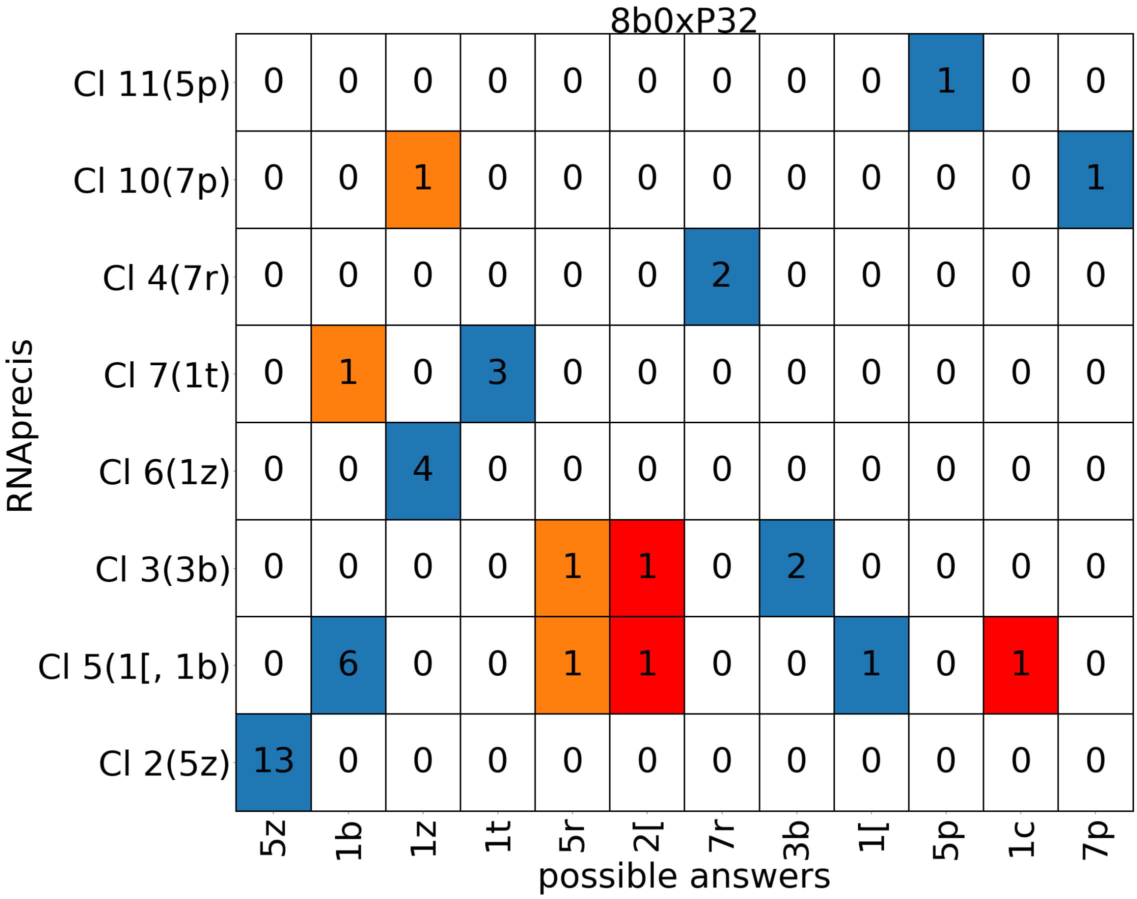

Fig AA: Classification matrix listing counts of probable sequence related conformation classes (horizontal axis) versus MINT-AGE clusters (vertical, with conformer classes in parentheses from MINT-AGE training, see Fig J) for the low detail training data set of sugar pucker-pair LDP32 with colors (from Section 3.4 in the main text): blue for match, orange for mismatch and red for pucker-pair mismatch. Columns listing multiple suite conformers are from sites solved in different conformations across sequence-related models, and any of the sequence-related conformations were accepted as a matching predictions. As pointed out in Section 3.4, the **1a**,**3g**,**8d** and **3d**,**7p** suites have multiple sequence-related conformations. This means that the counts in these two columns are inflated.

E.7 Results for the test data set 8b0xP23

8b0xP23: matches

name	p. answer	RNAprecis	p_{post}
8eiu_a-1964_a-1965	8d	**11**=(2g,8d)	0.79
7st2_1-1980_1-1981	8d	**5**=(8d,4d)	0.88
7st2_1-2614_1-2615	4g	**15**=(4g)	0.94
7st2_1-865_1-866	4g	**15**=(4g)	0.94
7st2_1-2296_1-2297	4a	**2**=(0a,#a,4a)	0.96
7st2_1-1668_1-1669	6g	**4**=(6g,4g)	0.99
7st2_1-1554_1-1555	2a	**1**=(2a)	1.0
7st2_1-2051_1-2052	4g	**4**=(6g,4g)	1.0
8eiu_a-2320_a-2321	6g	**4**=(6g,4g)	1.0
7st2_1-2867_1-2868	0a	**2**=(0a,#a,4a)	1.0
7st2_1-1344_1-1345	2a	**1**=(2a)	1.0
7st2_1-474_1-475	8d	**5**=(8d,4d)	1.0
7st2_1-1205_1-1206	2a	**1**=(2a)	1.0
7st2_1-2732_1-2733	2a	**1**=(2a)	1.0
7st2_1-442_1-443	8d	**5**=(8d,4d)	1.0
7st2_1-1653_1-1654	6j	**10**=(6j)	1.0
8eiu_a-60_a-61	2g	**11**=(2g,8d)	1.0
7st2_1-2645_1-2646	2a	**1**=(2a)	1.0
7st2_1-1996_1-1997	0i	**9**=(0i)	1.0
7st2_1-265_1-266	0i	**9**=(0i)	1.0

8b0xP23: pucker mismatches

name	p. answer	p_1	p_2	RNAprecis	p_{post}
7st2_1-227_1-228	4b	1.82	4.05	9=(0i)	0.94
7st2_1-1558_1-1559	6p	0.77	3.93	5=(8d,4d)	0.99
7st2_1-1210_1-1211	6p	0.91	4.34	11=(2g,8d)	1.0
7st2_1-322_1-323	2o	0.84	3.46	10=(6j)	1.0

8b0xP23 conformer mismatches

name	p. answer	RNAprecis	p_{post}	first match	p_{post}
7st2_1-2092_1-2093	6n	10=(6j)	1.0	3=(6n,0i,6j)	$6 \cdot 10^{-12}$
7st2_1-1020_1-1021	6n	2=(0a,#a,4a)	1.0	3=(6n,0i,6j)	$2 \cdot 10^{-54}$

Table M: Suites for which the RNAprecis predicted cluster matches the sequence related conformer (blue in the plots), the sugar pucker-pair determined by the Pperp criterion do not match the sugar pucker-pair of the sequence related conformers (red in the plots), or the RNAprecis predicted cluster does not match the sequence related conformer (orange in the plots); each sorted by posterior probability.

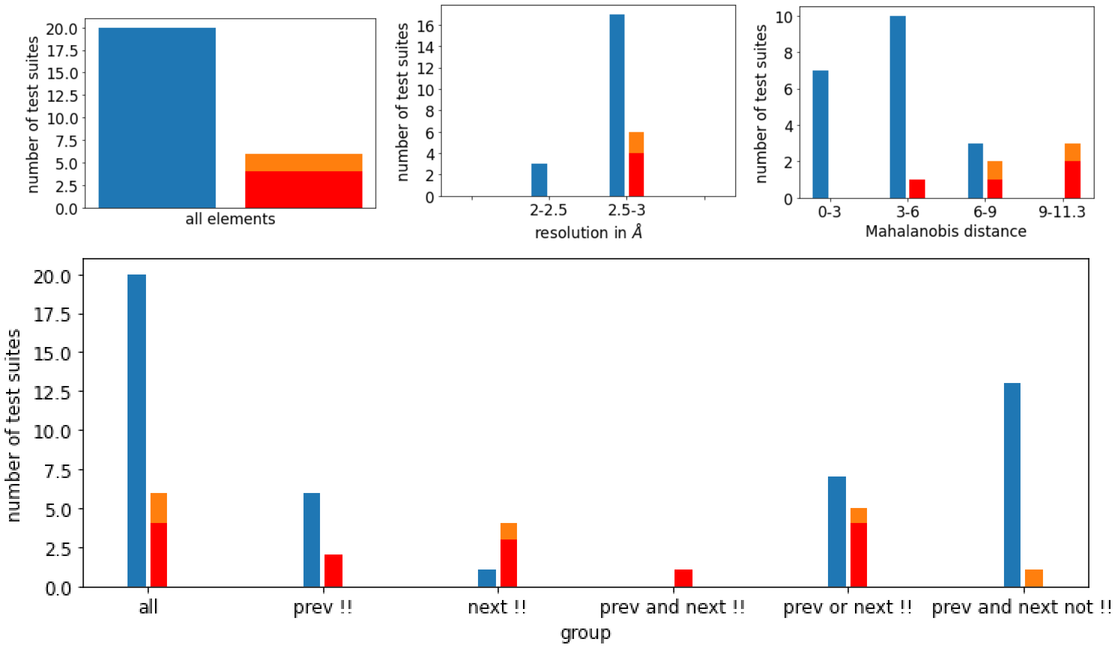

Fig AB: Relative distribution of agreements and disagreements of MINT-AGE cluster prediction with sequence related probable conformer classes for the test set LDT23 with color scheme (reflecting blue for match, orange for mismatch and red for pucker-pair mismatch) from Section 3.4 in the main text). Top left: overall histogram. Top middle: histogram reflecting varying resolutions of PDB suites. Top right: histogram reflecting varying Mahanalobis distances from the predicted cluster. One can see that with increasing distance the rate of mismatches increases. This indicates that despite its limitations discussed in Remark 2.3 in the main text the distance is useful as an empirical indicator of prediction confidence. Bottom: histogram also reflecting dependence on whether previous, next or both suites have been !! or not.

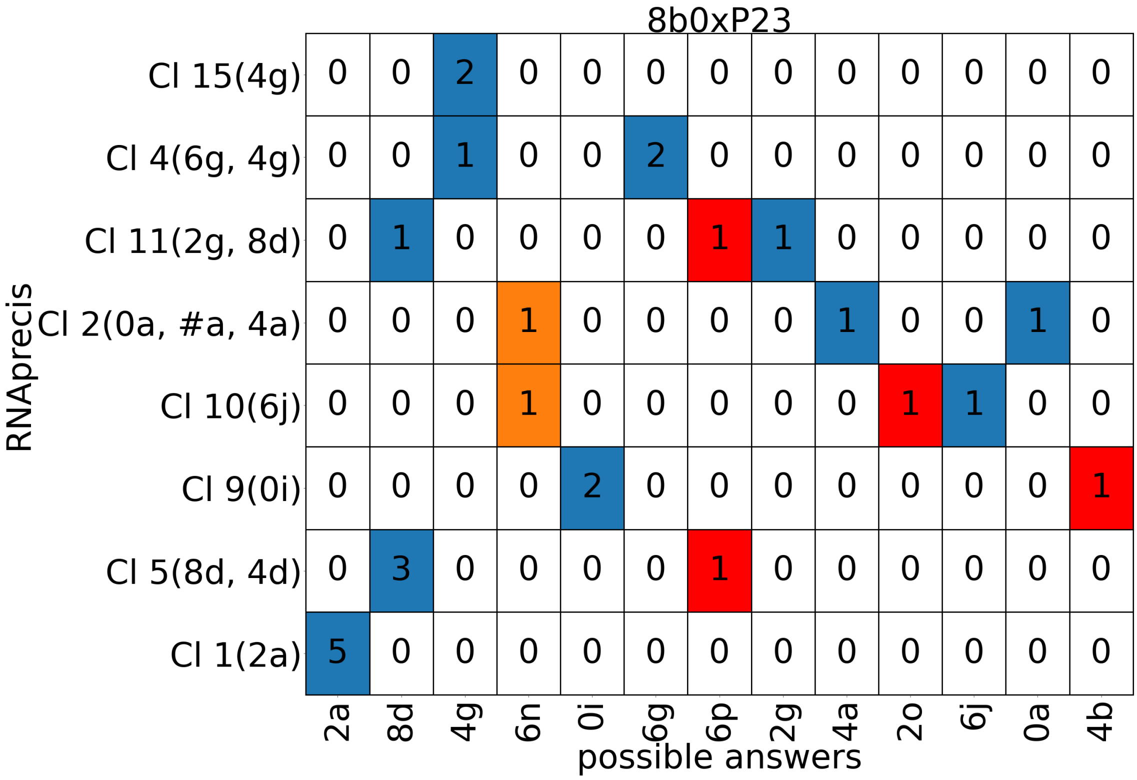

Fig AC: Classification matrix listing counts of probable sequence related conformation classes (horizontal axis) versus MINT-AGE clusters (vertical, with conformer classes in parentheses from MINT-AGE training, see Fig K) for the low detail training data set of sugar pucker-pair LDP23 with colors (from Section 3.4 in the main text): blue for match, orange for mismatch and red for pucker-pair mismatch. Columns listing multiple suite conformers are from sites solved in different conformations across sequence-related models, and any of the sequence-related conformations were accepted as a matching predictions. Columns listing multiple suite conformers are from sites solved in different conformations across sequence-related models, and any of the sequence-related conformations were accepted as a matching predictions.

E.8 Results for the test data set 8b0xP22

8b0xP22: matches

name	p. answer	RNAprecis	p_{post}
7st2_1-1130_1-1131	2z	**5**=(2z)	0.97
7st2_1-1247_1-1248	4p	**3**=(4p)	0.99
7st2_1-2249_1-2250	2u	**6**=(2u,2o,2[)	1.0
7st2_1-2344_1-2345	6p	**2**=(6p,!!)	1.0
7st2_1-370_1-371	2[	**1**=(2[)	1.0
7st2_1-321_1-322	2[	**1**=(2[)	1.0
7st2_1-1693_1-1694	2[	**1**=(2[)	1.0
7st2_1-829_1-830	6p	**2**=(6p,!!)	1.0
7st2_1-2021_1-2022	2[	**1**=(2[)	1.0

8b0xP22 conformer mismatches

name	p. answer	RNAprecis	p_{post}	first match	p_{post}
8eiu_a-1204_a-1205	6p	3=(4p)	0.68	2=(6p,!!)	0.32
7st2_1-1818_1-1819	4b	9=(0b)	1.0	7=(4b,0b)	$2 \cdot 10^{-3}$
7st2_1-788_1-789	4b	1=(2[)	1.0	4=(4b)	$4 \cdot 10^{-5}$
7st2_1-2866_1-2867	4s	3=(4p)	1.0	-	-
7st2_1-371_1-372	4s	3=(4p)	1.0	-	-

Table N: Suites for which the RNAprecis predicted cluster matches the sequence related conformer (blue in the plots), or the RNAprecis predicted cluster does not match the sequence related conformer (orange in the plots); each sorted by posterior probability. Since there are no cases where the sugar pucker-pair determined by the Pperp criterion do not match the sugar pucker-pair of the sequence related conformers (red in the plots) in this data set, this table is omitted.

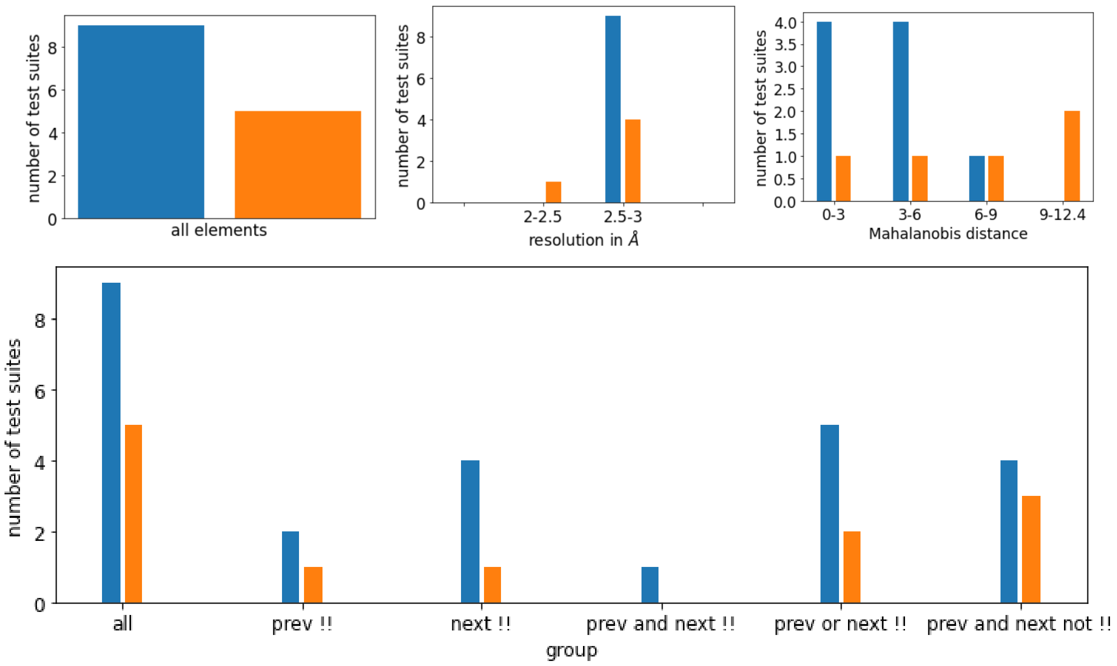

Fig AD: Relative distribution of agreements and disagreements of MINT-AGE cluster prediction with sequence related probable conformer classes for the test set LDT22 with color scheme (reflecting blue for match, orange for mismatch and red for pucker-pair mismatch) from Section 3.4 in the main text). Top left: overall histogram. Top middle: histogram reflecting varying resolutions of PDB suites. Top right: histogram reflecting varying Mahanalobis distances from the predicted cluster. One can see that with increasing distance the rate of mismatches increases. This indicates that despite its limitations discussed in Remark 2.3 in the main text the distance is useful as an empirical indicator of prediction confidence. Bottom: histogram also reflecting dependence on whether previous, next or both suites have been !! or not.

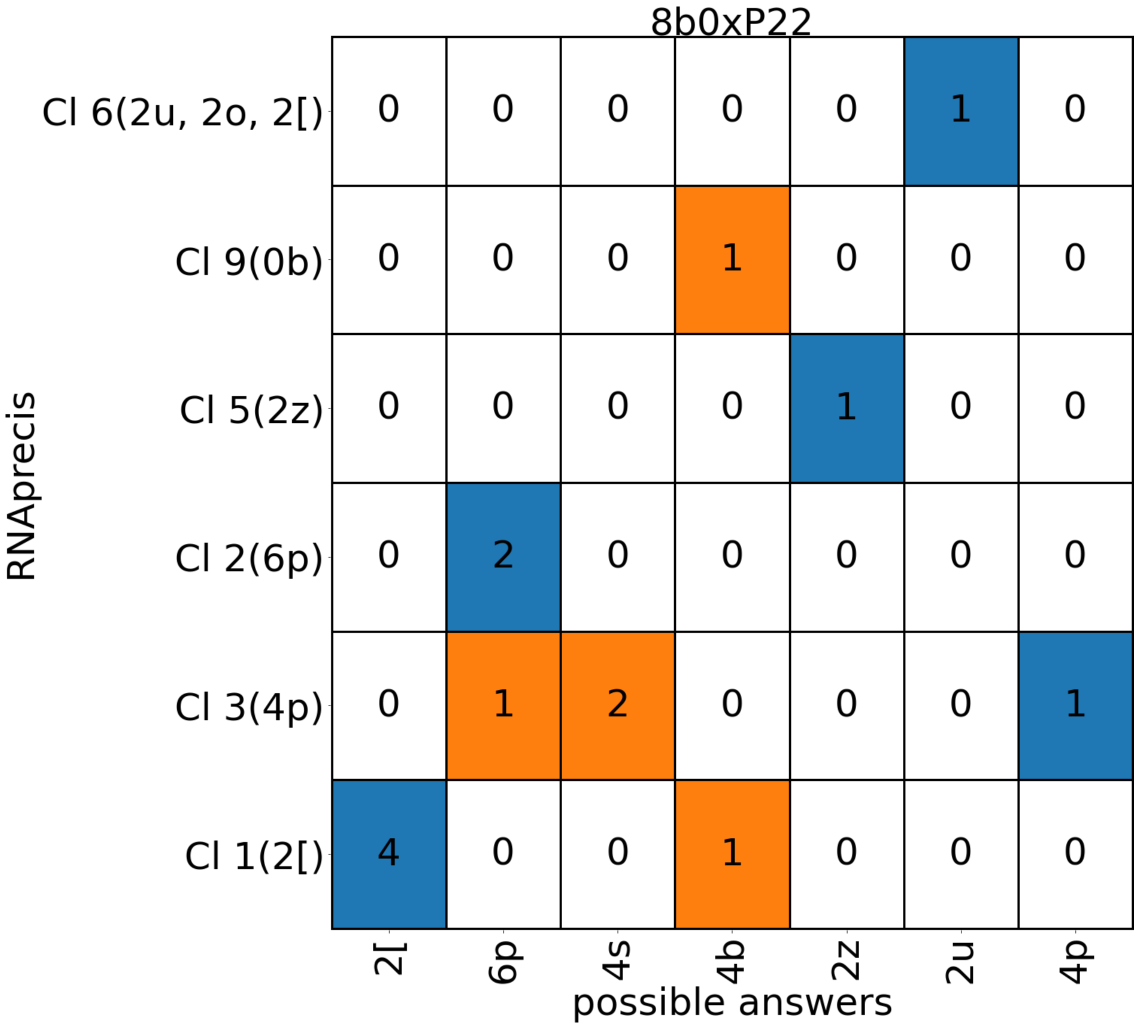

Fig AE: Classification matrix listing counts of probable sequence related conformation classes (horizontal axis) versus MINT-AGE clusters (vertical, with conformer classes in parentheses from MINT-AGE training, see Fig L) for the low detail training data set of sugar pucker-pair LDP22 with colors (from Section 3.4 in the main text): blue for match, orange for mismatch and red for pucker-pair mismatch. Columns listing multiple suite conformers are from sites solved in different conformations across sequence-related models, and any of the sequence-related conformations were accepted as a matching predictions. Columns listing multiple suite conformers are from sites solved in different conformations across sequence-related models, and any of the sequence-related conformations were accepted as a matching predictions.

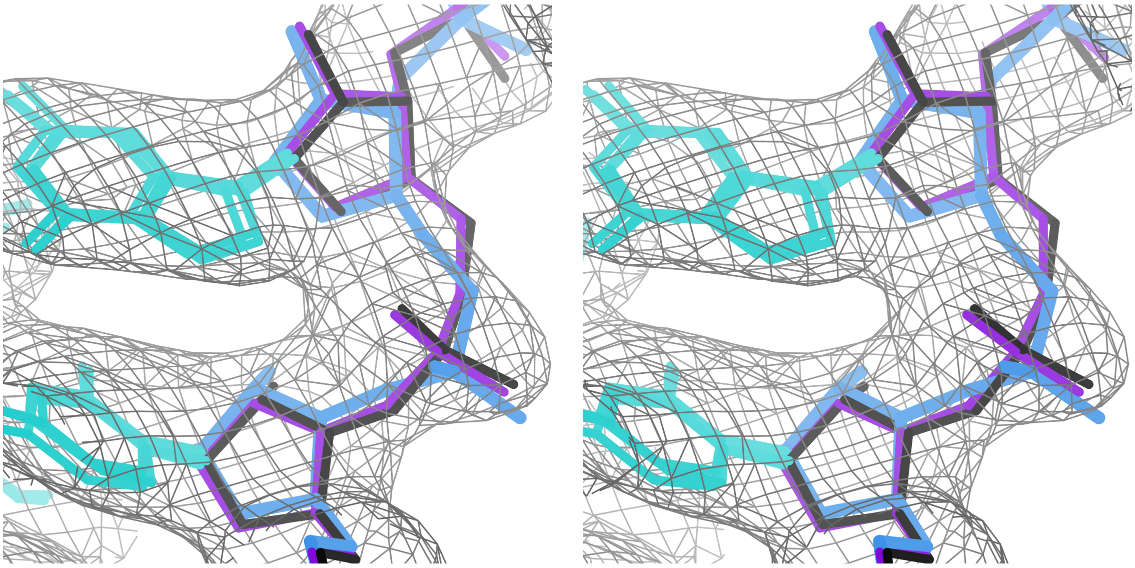

Fig AF: A stereo image, showing a comparison of 5fk1, suite 68, with the original deposited !! conformation (black backbone), manually corrected (sky blue backbone), and after refinement (purple backbone) in Phenix. RCrane was used in Coot to switch the conformation to a 1b conformation, as suggested by RNAprecis and matching the corresponding conformation in the reference structure (5fjc, suite 68). In terms of large ($> 18°$) backbone dihedral changes, ϵ went from 218° to 257° (+39°), α went from 148° to 71° ($-77°$), and γ went from 164° to 224° (+60°). Refinement reverted the correction to the original !! conformation, even though the map does not have sufficient detail to justify a non-standard conformation. For the $\epsilon/\alpha/\gamma$ dihedrals, they reverted to 229°/137°/176°, respectively. Analogous to cases of protein sidechain rotamer outliers in ambiguous density [1], additional restraints or refinement weight adjustments seem to be needed to help fix appropriate RNA backbone conformations in areas with less data.

References

[1] Headd JJ, Immormino RM, Keedy DA, Emsley P, Richardson DC, Richardson JS. Autofix for backward-fit sidechains: using MolProbity and real-space refinement to put misfits in their place. Journal of structural and functional genomics. 2009;10(1):83–93.
